# Supplementary material for: Tyrosine phosphorylation of both STAT5A and STAT5B is necessary for maximal IL-2 signaling and T cell proliferation
Source: Nat Commun. 2024 Aug 27;15:7372. doi: 10.1038/s41467-024-50925-6 (PMC11349758; doi:10.1038/s41467-024-50925-6)
Supplement: Supplementary file 1 — Supplementary Information [file 41467_2024_50925_MOESM1_ESM.pdf]

Tyrosine phosphorylation of both STAT5A and STAT5B is necessary for maximal IL-2 signaling and T cell proliferation

Jian-Xin Lin[1,#,*], Meili Ge[1,#,5], Cheng-yu Liu[2], Ronald Holewinski[3], Thorkell Andresson[3], Zu-Xi Yu[4], Tesfay Gebregiorgis[1], Rosanne Spolski[1], Peng Li[1,6], and Warren J. Leonard[1,*]

[1]Laboratory of Molecular Immunology and Immunology Center

National Heart, Lung, and Blood Institute

National Institutes of Health

Bethesda, MD 20817-1694

[2]Transgenic Mouse Core Facility

National Heart, Lung, and Blood Institute

National Institutes of Health

Bethesda, MD 20892-8018

[3]Leidos Biomedical Research, Inc.

Frederick National Laboratory for Cancer Research

Frederick, MD 21701

[4]Pathology Core, National Heart Heart, Lung, and Blood Institute

National Institutes of Health

Bethesda, MD 20892

[#]Contributed equally
[*]Correspondence should be sent to linjx@nhlbi.nih.gov or wjl@helix.nih.gov
[5]Current address: State Key Laboratory of Experimental Hematology
Institute of Hematology & Blood Diseases Hospital
Chinese Academy of Medical Sciences & Peking Union Medical College,
Tianjin 300020, People's Republic of China.
[6]Current address: Amgen, Inc., 2301 Research Blvd., Rockville, MD 20850

Supplementary Figure 1

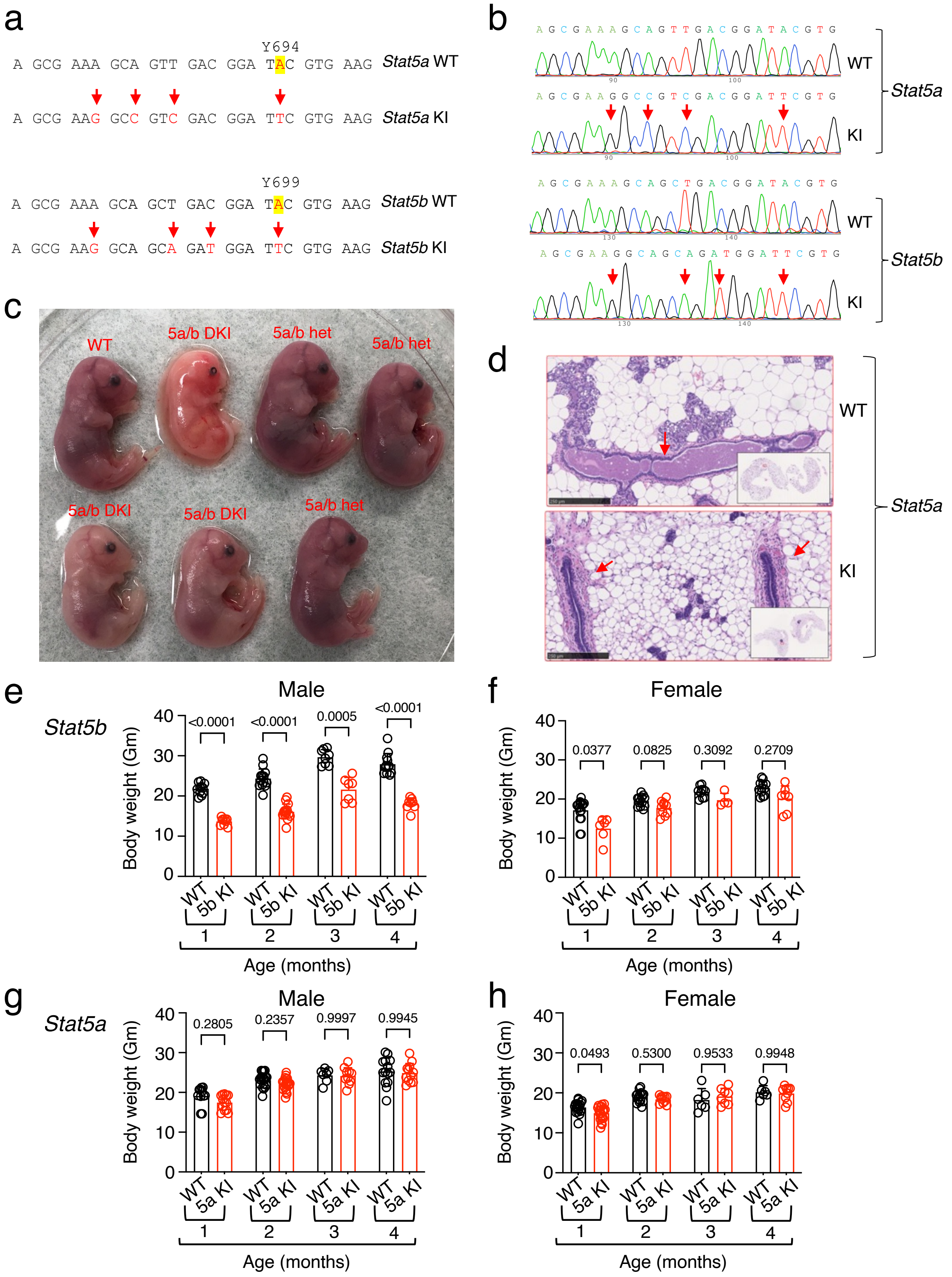

Supplementary Fig. 1. Generation of STAT5A^{Y694F} (*Stat5a* KI) and STAT5B^{Y699F} (*Stat5b* KI) mutant mice by CRISPR/Cas9, with defective of mammary gland development in *Stat5a* KI female mice and lower body weight in *Stat5b* KI male mice. **(a)** Design of *Stat5a* and *Stat5b* TAC (tyrosine) to TTC (phenylalanine) mutations (red arrows and letters in red) for generating the *Stat5a* KI and *Stat5b* KI mice. To prevent re-cutting by Cas9 after repair, 3 additional nucleotides (indicated by red arrows and red letters) 5' of the TAC were also changed, without altering the encoded amino acids. (**b**) Correct mutations were confirmed by Sanger sequencing. (**c**) A set of fetuses produced by a *Stat5a/Stat5b* DKI heterozygous female mouse and their genotypes wild-type (WT), *Stat5a/Stat5b* DKI homozygous (5a/b KI), and *Stat5a*/*b* DKI heterozygous (5a/b het) were determined by Sanger sequencing. (**d**) H&E stains of mammary gland sections 1 day before parturition. Enlarged fat cells and alveolar duct filled with milk were evident in a well-developed mammary gland in a WT female mouse (top panel, red arrow) and small fat cells and empty alveolar duct in a *Stat5a* KI female mouse (bottom panel, red arrows). (**e-h**) Weights of male (**e** and **g**) and female (**f** and **h**) WT and *Stat5b* KI (**e** and **f**) and WT and *Stat5a* KI (**g** and **h**) mice. In panel **e,** n=11 for 1-month-old, n=13 for 2-month-old, n=8 for 3-month-old, and n=12 for 4-month-old *Stat5b* WT male mice; n=9 for 1-month-old, n=14 for 2-month-old and n=7 for 3-month-old, and n=8 for 4-month-old *Stat5b* KI male mice. In panel **f**, n=16 for 1-month-old, n= 13 for 2-month-old, n=10 for 3-month-old, and n=12 for 4-month-old *Stat5b* WT female mice; n=6 for 1-month-old, n=10 for 2-month-old, n=4 for 3-month-old, and n=7 for 4-month-old *Stat5b* KI female mice. In panel **g**, n=11 for 1 month-old, n=19 for 2-month-old, n=7 for 3-month-old, and n=14 for 4-month-old *Stat5a* WT male mice; n=13 for 1 month-old, n=19 for 2-month-old, n=10 for 3-month-old, and n=12 for 4-month-old *Stat5a* KI male mice. In panel **h**, n=17 for 1 month-old, n=15 for 2-month-old, n=6 for both 3 month and 4-month-old *Stat5a* WT female mice; n=20 for 1-month-old, n=10 for 2-month-old, n=8 for 3-mon-old, and n=9 for 4-month-old *Stat5a* KI female mice. In panels **e-h**, error bars (SD) and p values are shown, which were determined by multiple unpaired *t* test using two-stage step-up method of Benjamini, Krieger and Yekutieli; WT and mutant mice shown in black and red open bars, respectively.

Supplementary Figure 2

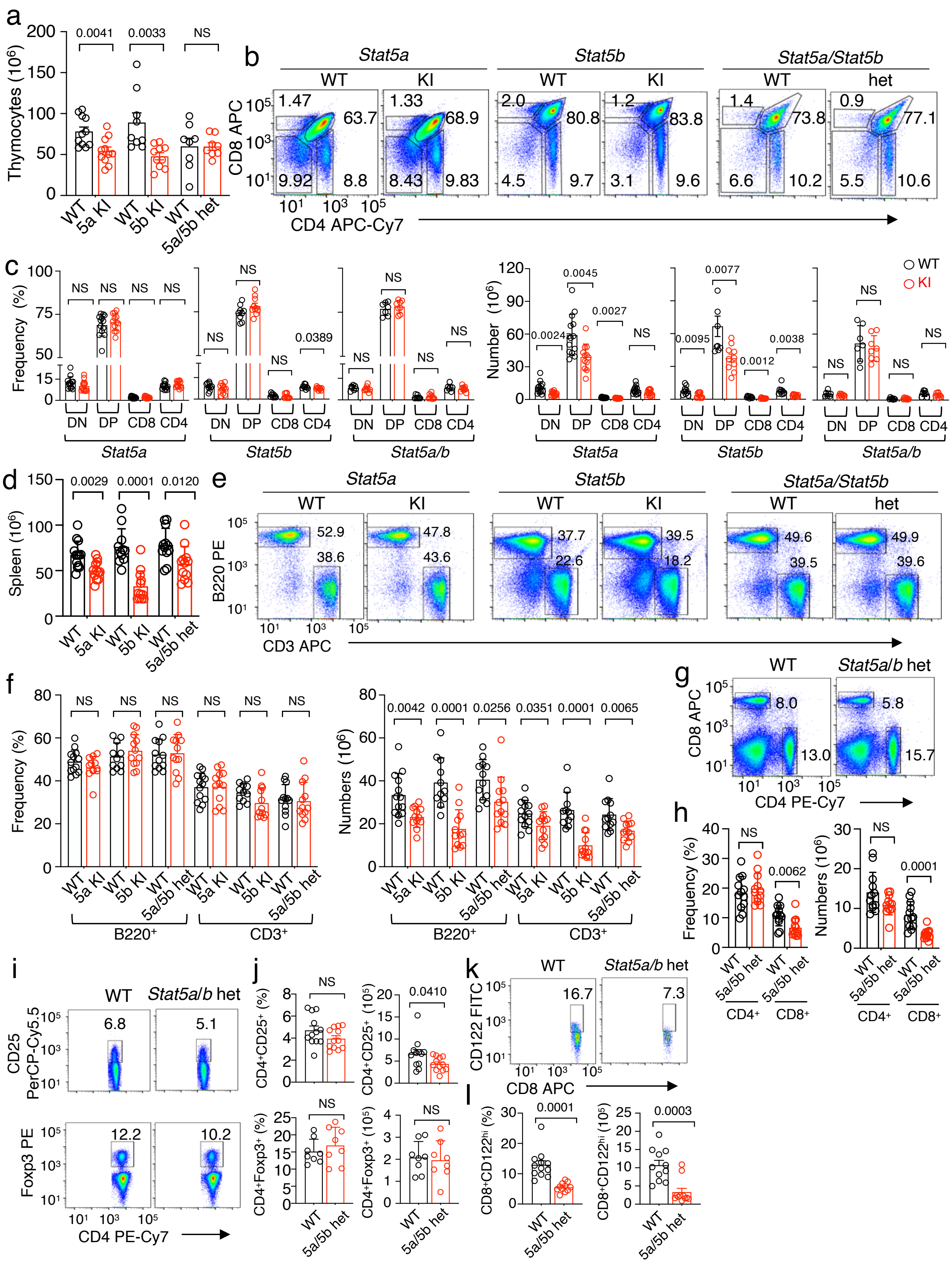

Supplementary Fig. 2. Diminished thymic and/or splenic T cell numbers in *Stat5a* KI, *Stat5b* KI, and *Stat5a*/*Stat5b* DKI heterozygous mice. (**a**) Total thymocyte numbers in WT versus *Stat5a* KI homozygous (n=11 for *Stat5a* WT and *Stat5a* KI, respectively), *Stat5b* KI homozygous (n=9 for *Stat5b* WT and n=10 for *Stat5b* KI), and *Stat5a/Stat5b* DKI heterozygous (n=8 for both WT and mutant) mice. (**b**) Representative flow cytometric profiles of WT (left panels) and *Stat5a* KI homozygous, *Stat5b* KI homozygous, and *Stat5a/Sta5b* DKI heterozygous (right panels) thymocytes stained with anti-CD4 and anti-CD8. (**c**) Frequency (3 left panels) and numbers (3 right panels) of WT $CD4^{-}CD8^{-}$ (DN), $CD4^{+}CD8^{+}$ double positive (DP), $CD8^{+}$ single positive (CD8), and $CD4^{+}$ single positive (CD4) thymocytes in *Stat5a* KI homozygous (n= 12 for *Stat5a* WT and n=11 for *Stat5a* KI), *Stat5b* KI homozygous (n=9 for *Stat5b* WT and n=10 for *Stat5b* KI), and *Stat5a/Stat5b* DKI heterozygous (n=7 for *Stat5a*/*Stat5b* WT and n=8 for *Stat5a*/*Stat5b* het) mice. (**d**) Summary of total spleen cell numbers (n=13 for *Stat5a* WT, n=12 for *Stat5a* KI, n=11 for *Stat5b* WT, n=12 for *Stat5b* KI, n=12 for *Stat5a*/*Stat5b* WT, and n=12 for *Stat5a*/*Stat5b* het) in the indicated mice. (**e**) Representative flow cytometric profiles of splenic $B220^{+}$ and $CD3^{+}$ cells in WT and corresponding *Stat5a* KI homozygous, *Stat5b* KI homozygous, and *Stat5a/Stat5b* DKI heterozygous mice. (**f**) Frequency (left graph) and numbers (right graph) of $B220^{+}$ and $CD3^{+}$ splenocytes in WT (black open bars, n=13 for *Stat5a*, n=11 for *Stat5b*, and n=12 for *Stat5a*/*Stat5b*) and *Stat5a* KI homozygous (n=12), *Stat5b* KI homozygous (n=12), and *Stat5a/Stat5b* DKI heterozygous (n=12) (red open bars) mice. (**g** and **h**) Representative flow cytometric profiles (**g**) and frequency and numbers (**h**) of WT (left panel, n=12) and *Stat5a*/*Stat5b* DKI heterozygous (right panel, n=12) splenic $CD4^{+}$ and $CD8^{+}$. In **h**, WT data are in black and *Stat5a*/*Stat5b* DKI heterozygous data in red. (**i** and **j**) Representative flow cytometric profiles (**i**) of $CD4^{+}CD25^{+}$ (top panels) and $CD4^{+}Foxp3^{+}$ (bottom panels) in WT (left panels) and *Stat5a/Stat5b* DKI heterozygous (right panels) mice. (**j**) Frequencies (top left bar graph) and numbers (top right bar graph) of $CD4^{+}CD25^{+}$, $CD4^{+}Foxp3^{+}$ (bottom bar graphs) cells in WT (n=12) and *Stat5a/Stat5b* DKI heterozygous (n=12) mice. (**k**) Representative flow cytometric profiles of $CD8^{+}CD122^{hi}$ cells in WT and *Stat5a/Stat5b* DKI het mice. The gating strategies for panel **b** are shown in **Supplementary**

Fig. 8a and panels **e**, **g**, **i**, and **k** are shown in **Supplementary Fig. 8b**. (**l**) Frequency (left graph) and numbers (right graph) of $CD8^+CD122^{hi}$ cells in WT (n=11) and *Stat5a*/*Stat5b* DKI heterozygous (n=11) mice. Panels **c** (3 left bar graphs), **f** (left bar graph), **h** (left bar graph), **j** (left bar graphs), and **l** (left bar graph) are the percentage of the gated cells, panels **a, c (**three right bar graphs**)**, **d**, **f** (right bar graph)**, h** (right bar graph), **j** (right bar graphs)**,** and **l** (right bar graph) are the number of the gated cells. The black and red open bars represent WT and mutant samples, respectively. In panels **a**, **c**, **d**, **f**, and **h**, error bars (SD) and adj p-values are shown, which were determined by multiple unpaired *t* tests using two-stage step-up method of Benjamini, Krieger and Yekutieli; in panels **j** and **l,** error bars (SD) and p values are shown, which were determined using unpaired and two-tailed *t* test.

Supplementary Figure 3

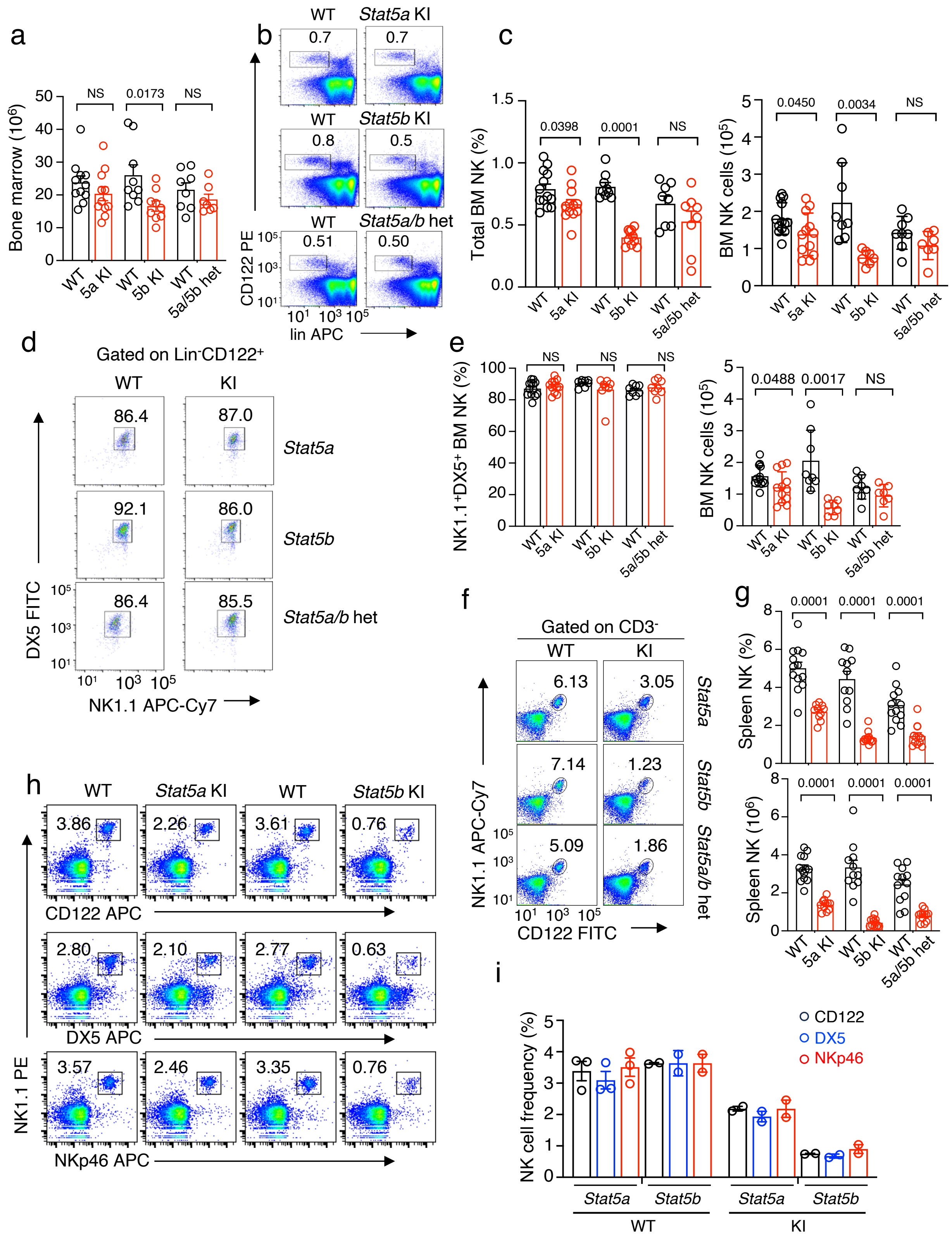

Supplementary Fig. 3. Decreased frequency and numbers of bone marrow NK cells in *Stat5b* KI mice and lower frequency and numbers of splenic NK cells in *Stat5a* KI, *Stat5b* KI, and *Stat5a/Stat5b* DKI het mice. (**a**) Numbers of total bone marrow cells harvested from both femurs from each WT (n=11 for *Stat5a* WT, n=9 for *Stat5b* WT, and n=8 for *Stat5a*/*Stat5b* WT), *Stat5a* KI homozygous (n=11), *Stat5b* KI homozygous (n=9), and *Stat5a/Stat5b* DKI heterozygous (n=7) mice. (**b**) Representative flow cytometric profiles of WT (left panels) and *Stat5a* KI homozygous, *Stat5b* KI homozygous, and *Stat5a/Stat5b* DKI heterozygous (right panels) total bone marrow cells gated on total BM NK cells (lin^-CD122^+). (**c**) Bar graph of the frequency (left graph) and number (right graph) of total BM NK cells in WT (black bars, n=13 for *Stat5a* WT, n=8 for both *Stat5b* WT and *Stat5a*/*Stat5b* WT) and each mutant mouse line (red bars, n=12 for *Stat5a* KI, n=7 for both *Stat5b* KI and *Stat5a*/*Stat5b* het). (**d**) Representative flow cytometric profiles of BM mature NK cells (lin^- $CD122^+DX5^+NK1.1^+$) in WT (left panels), and *Stat5a* KI homozygous, *Stat5b* KI homozygous, and *Stat5a*/*Stat5b* DKI heterozygous mice (right panels). (**e**) Bar graphs of the frequency (left graph) and numbers (right graph) of BM mature NK cells in WT (black bars, n=13 for *Stat5a* WT, n=8 for both *Stat5b* WT and *Stat5a*/*Stat5b* WT) and each mutant mouse line (red bars, n=12 for *Stat5a* KI, n=7 for both *Stat5b* KI and *Stat5a*/*Stat5b* het). (**f**) Representative flow cytometric profiles of total splenic NK cells ($CD3^-CD122^+NK1.1^+$) in WT (left panels), *Stat5a* KI (top right panel), *Stat5b* KI (middle right panel), and *Stat5a/Stat5b* DKI heterozygous (bottom right panel) mice. (**g**) Bar graphs of the frequencies (left graph) and numbers (right graph) of total splenic NK cells in WT (black bars, n=13 for *Stat5a* WT, n=11 for *Stat5b* WT, and n=12 for *Stat5a*/*Stat5b* WT) and *Stat5a* KI, *Stat5b* KI, and *Stat5a/Stat5b* DKI heterozygous (red bars, n=11 for *Stat5a* KI, n=12 for both *Stat5b* KI and *Stat5a*/*Stat5b* het) mice. The numbers in panels **b**, **d**, **f,** and **h** are percentage of gated cells; in panels **a**, **c**, **e**, and **g,** error bars (SD) and p-values are shown, which were determined by multiple unpaired *t* test using two-stage step-up method of Benjamini, Krieger and Yekutieli. (**h**) Representative flow cytometric profiles of spleen NK cells gated on $CD3^-$ cells, then on NK1.1 with anti-CD122 (top row), anti-CD49b (DX5, middle row), or anti-NKp46 (CD335, bottom row) in WT, *Stat5a* KI, and *Stat5b* KI mice. (**i**) Summary of frequency of $NK1.1^+CD122^+$, $NK1.1^+DX5^+$, and $NK1.1^+NKp46^+$ spleen NK cells from WT (n=3

for *Stat5a* WT and n=2 for *Stat5b* WT), *Stat5a* KI (n=2), and *Stat5b* KI (n=2) mice and the error bars (SEM) are shown. The gating strategies for panels **b**, **d**, **f**, and **h** are shown in **Supplementary Fig. 11**.

Supplementary Figure 4

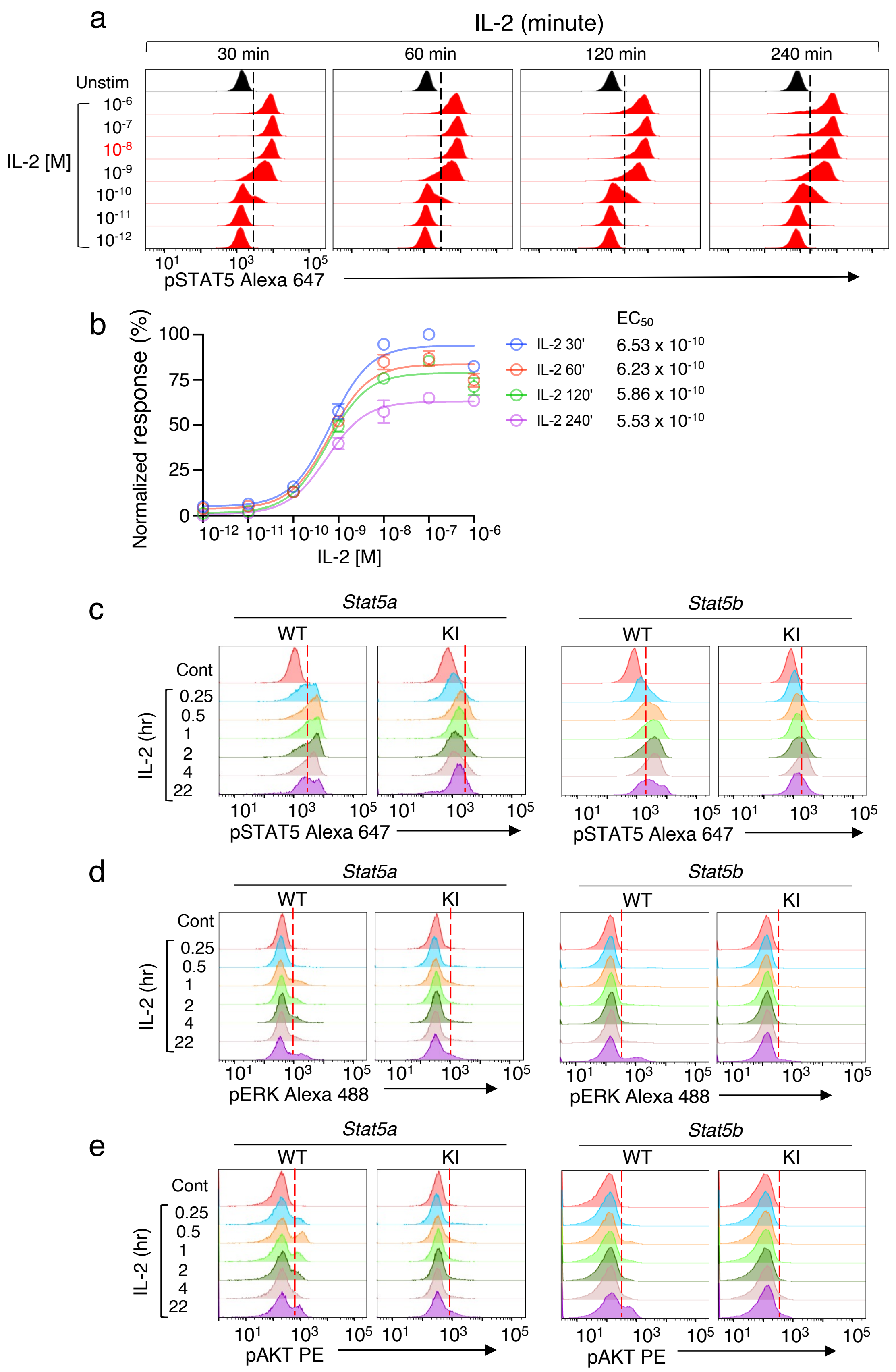

Supplementary Fig. 4. Diminished activation of pSTAT5, pERK, and pAKT by IL-2 in *Stat5a* KI and *Stat5b* KI CD8$^+$ T cells. (**a**) Representative flow cytometric profiles of time-course and dose response of pYSTAT5 in freshly isolated CD8$^+$ T cells stimulated by IL-2 (n=2). (**b**) Shown are normalized data and EC_{50} for each dose of IL-2 that were determined by a nonlinear regression model ([Agonist] vs. Response (three parameters)). (**c**-**e**) Representative flow cytometric profiles of two time-course experiments showing the levels of pSTAT5 (**c**), pERK (**d**), and pAKT (**e**) in WT, *Stat5a* KI (n=2), and *Stat5b* KI (n=2) CD8$^+$ T cells stimulated by IL-2 as indicated. The gating strategies for panels **a, c, d,** and **e** are shown in **Supplementary Fig. 12a**.

Supplementary Fig. 5

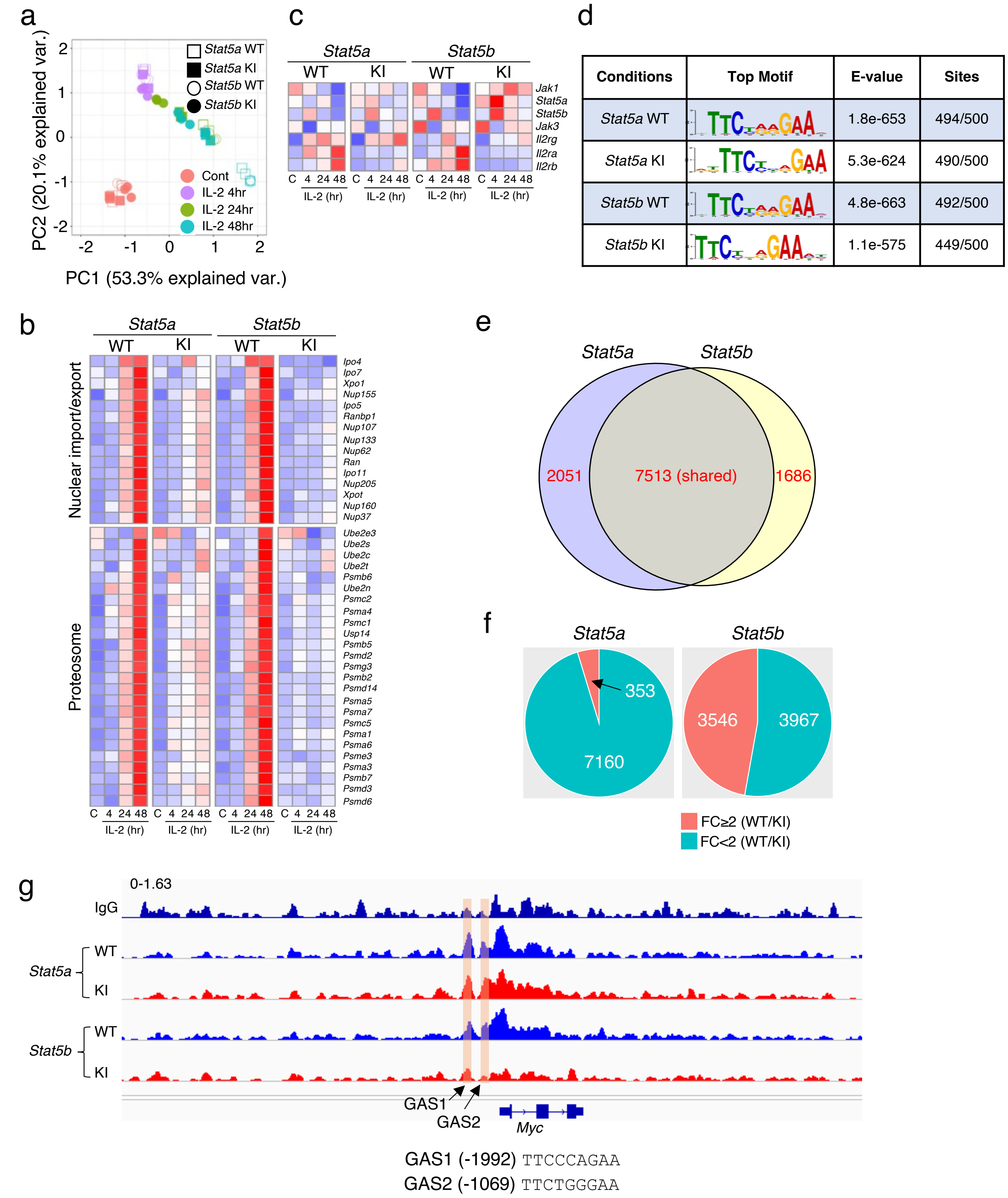

Supplementary Fig. 5. Decreased IL-2-induced mRNA expression and STAT5 binding in *Stat5* KI CD8+ T cells. (**a**) Principal component analysis of mRNAs expressed in WT (open squares and open circles, *Stat5a* KI (filled squares), and *Stat5b* KI (filled circles) CD8+ T cells stimulated by IL-2 for 4 hrs (red), 24 hrs (green), and 48 hrs (arctic). The PCA shown used dimension reduction analysis. (**b**) Heatmap showing dysregulated mRNAs in *Stat5a* KI and *Stat5b* KI cells encoding proteins involved in Nuclear import/export and the Proteosome. (**c**) Heatmap showing IL-2-induced expression levels of mRNAs for *Jak1*, *Stat5a*, *Stat5b*, *Jak3*, *Il2rg*, *Il2ra*, and *Il2rb*, in WT and *Stat5* KI CD8+ T cells. (**d**) ChIP-seq analysis showing the consensus GAS motifs being the top motifs in WT and *Stat5* KI cells. (**e**) Venn diagram showing the numbers of STAT5 binding peaks overlapped in *Stat5a* WT and *Stat5b* WT cells. (**f**) Pie graphs showing the number of STAT5 binding peaks with FC ≥ 2 (in crimson) and FC < 2 (in cyan) in WT vs. *Stat5a* KI and WT vs. *Stat5b* KI cells. (**g**) IGV tracks showing STAT5 binding to the *Myc* locus at 2 GAS motifs. The numbers on the bar charts show normalized data range for the locus.

Supplementary Figure 6

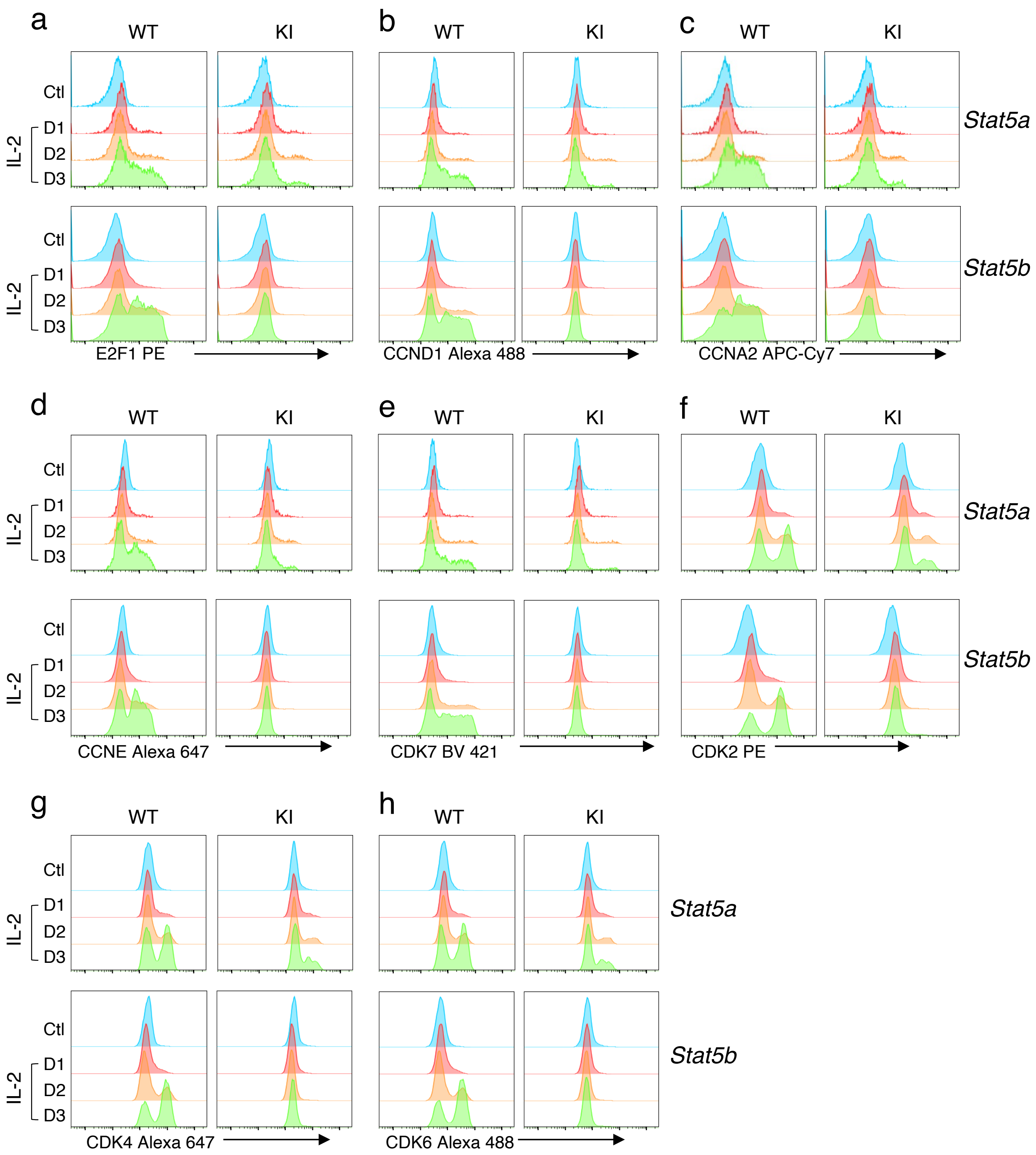

Supplementary Fig. 6. Decreased IL-2-induced expression of E2F1, CCND1, CCNA2, CCNE, CDK7, CDK2, CDK4, and CDK6 in *Stat5a* and *Stat5b* KI CD8$^+$ T cells. (**A-H**) Representative flow cytometric profiles of two experiments for E2F1 (**a**), CCND1 (**b**), CCNA2 (**c**), CCNE (**d**), CDK7 (**e**), CDK2 (**f**), CDK4 (**g**), and CDK6 (**h**). In each panel, data for *Stat5a* WT and KI are in the upper two plots and those for *Stat5b* are in

the lower two plots. Shown are control unstimulated cells (Ctl) and cells stimulated with IL-2 for 1 day, 2 days, and 3 days (D1, D2, and D3). The gating strategies for panels **a, b, c, d,** and **e** are shown in **Supplementary Fig. 12b** and panels **f**, **g**, and **h** are shown in **Supplementary Fig. 12c**.

Supplementary Figure 7

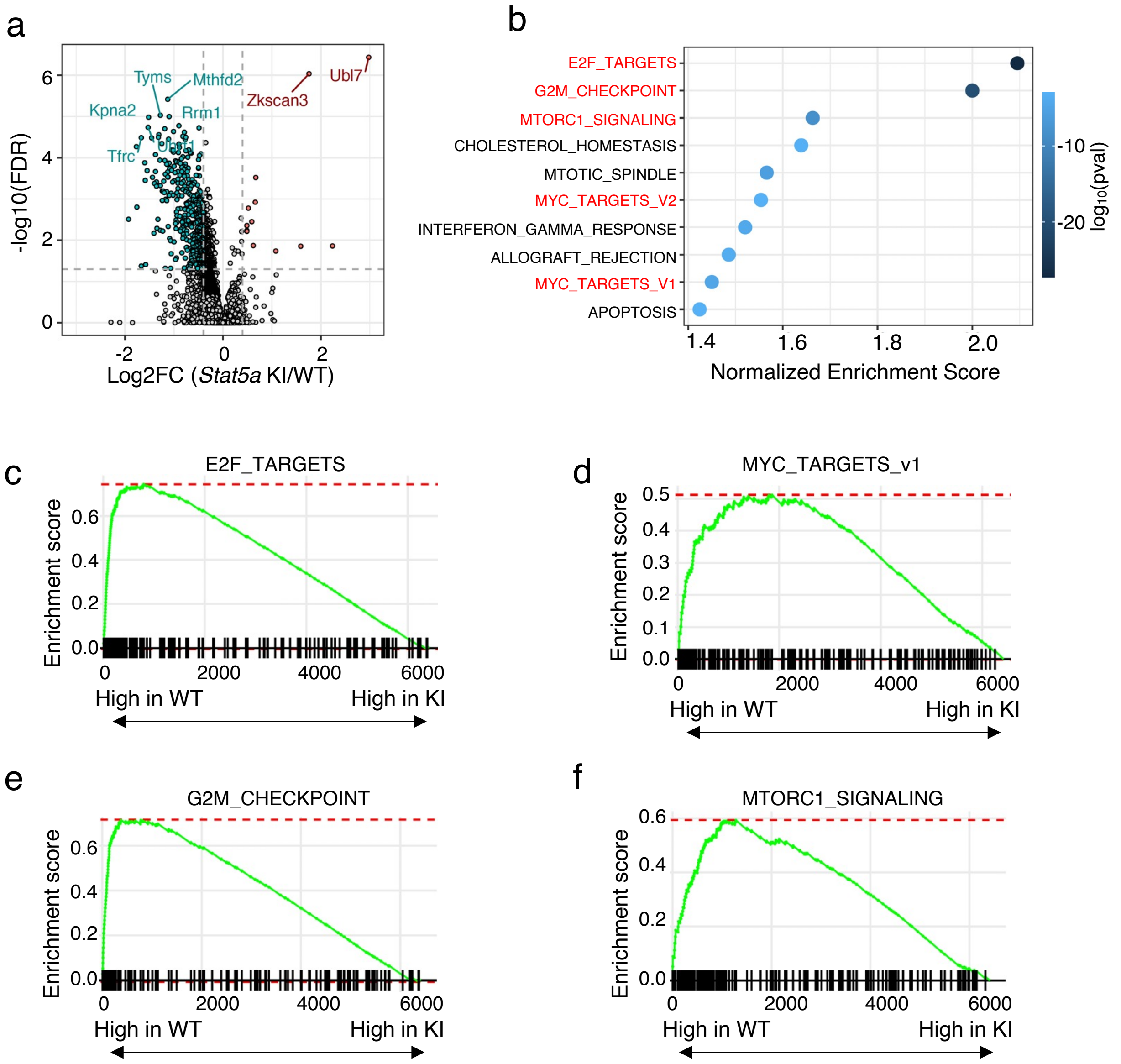

Supplementary Fig. 7. Identification of dysregulated IL-2-induced proteins in *Stat5a* KI CD8+ T cells by global proteomics analysis. (**a**) Scatter plot showing dysregulated proteins in *Stat5a* KI as compared to WT CD8+ T cells in response to IL-2 stimulation. (**b**) Top 10 differentially regulated Hallmark genesets identified by GSEA. (**c** to **f**) Enrichment of E2F_TARGETS (**c**), MYC-TARGETs_v1 (**d**), G2M_CHECKPOINT (**e**), and MTORC1_SIGNALING (**f**) genesets in WT vs. KI CD8+ T cells stimulated with IL-2.

Supplementary Figure 8

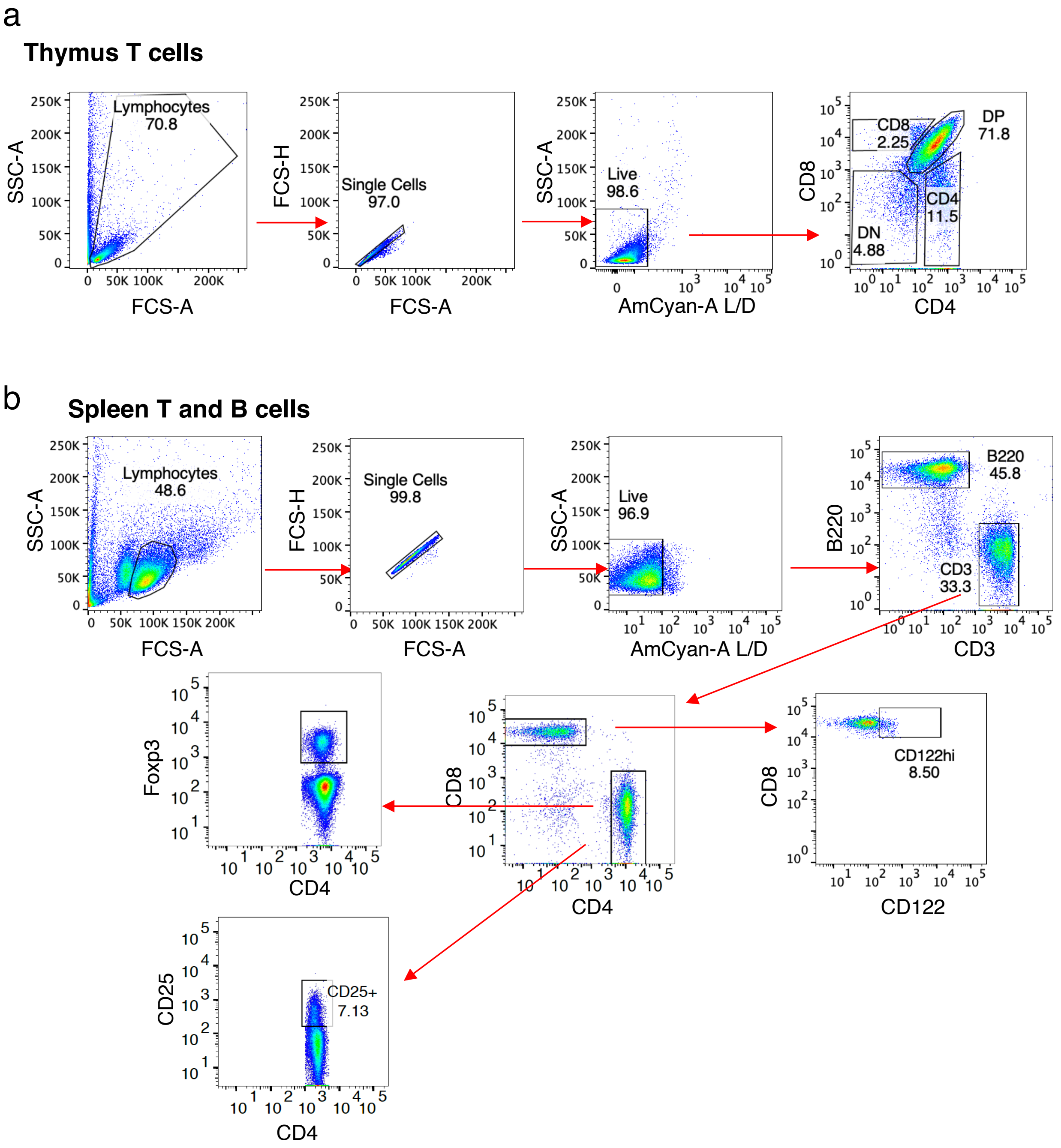

Supplementary Fig. 8. Flow cytometry gating strategies for thymic T cells and splenic T and B cells. (**a**) Gating strategies for flow cytometry analysis of thymic T (**Supplementary Fig. 2b**). (**b**) Gating strategies for flow cytometry analysis of splenic T and B cells (**Fig. 2a, c, e, g,** and **i, Supplementary Fig. 2e, g, i,** and **k**).

Supplementary Figure 9

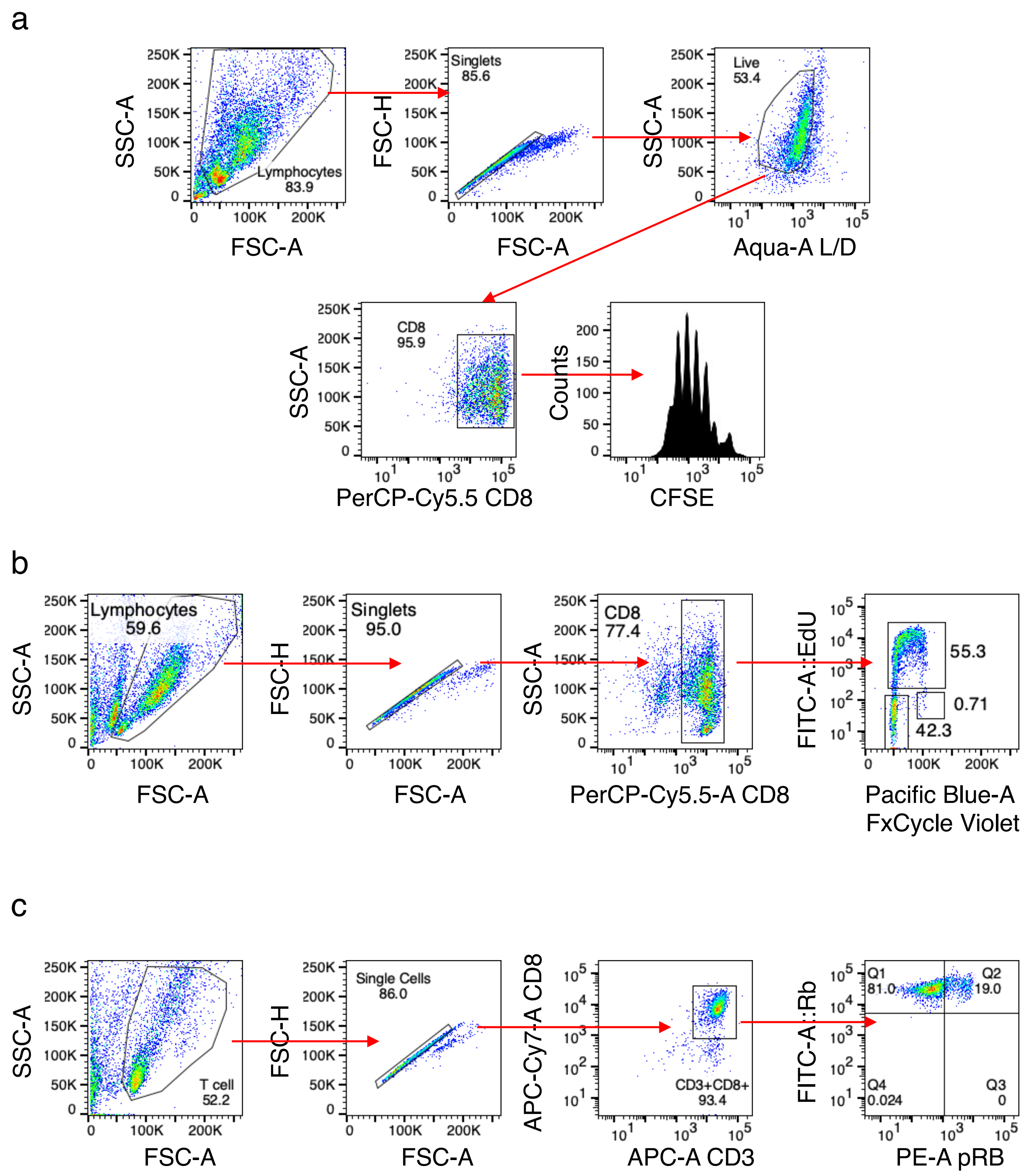

Supplementary Fig. 9. Flow cytometry gating strategies for IL-2-induced (**a**) proliferation, (**b**) cell cycle analysis, and (**c**) pRB expression in freshly isolated $CD8^+$ T cells. Gating strategies for **Fig. 3a, c, f,** and **g**).

Supplementary Figure 10

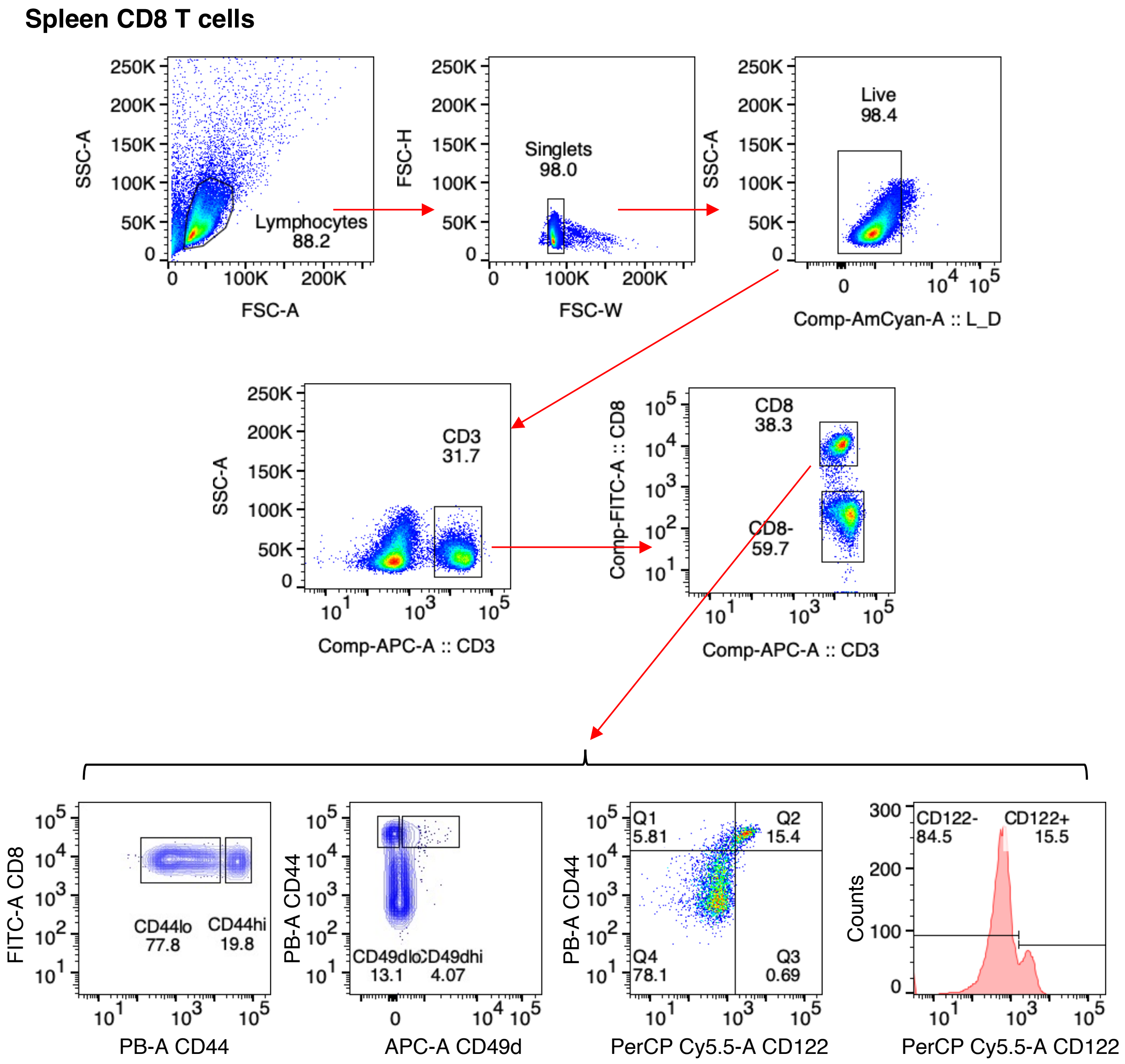

Supplementary Fig. 10. Flow cytometry gating strategies for splenic CD8$^+$ T cells. Gating strategies for flow cytometry analysis of spleen CD8$^+$ T cells (**Fig. 4d, f, h,** and **j**).

Supplementary Figure 11

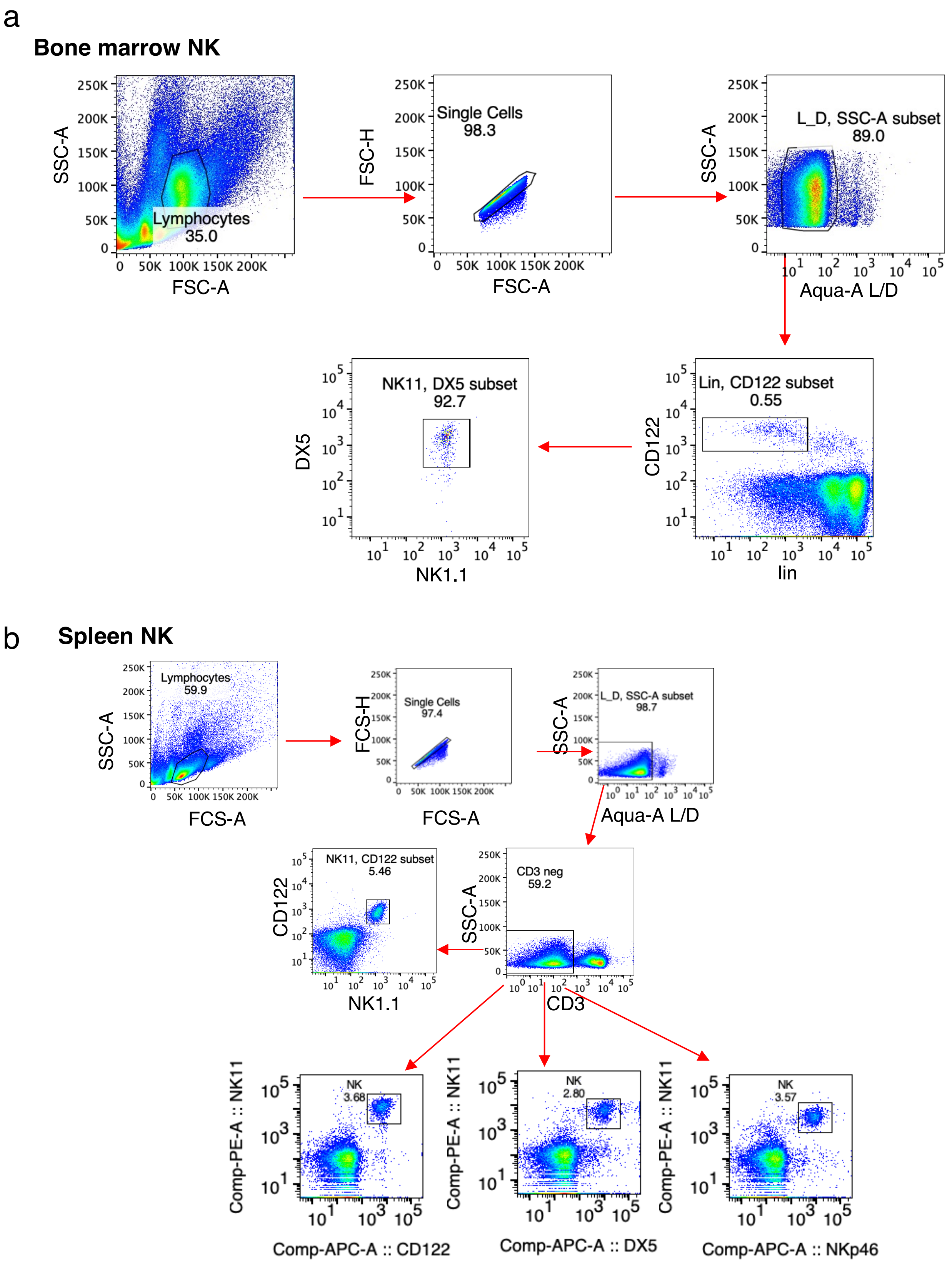

Supplementary Fig. 11. Flow cytometry gating strategies for bone marrow and splenic NK cells. (**a**) Gating strategies for flow cytometry analysis of bone marrow NK cells (**Supplementary Fig. 3b** and **3d**). (**b**) Gating strategies for flow cytometry analysis of splenic NK cells (**Supplementary Fig. 3f** and **h**).

Supplementary Figure 12

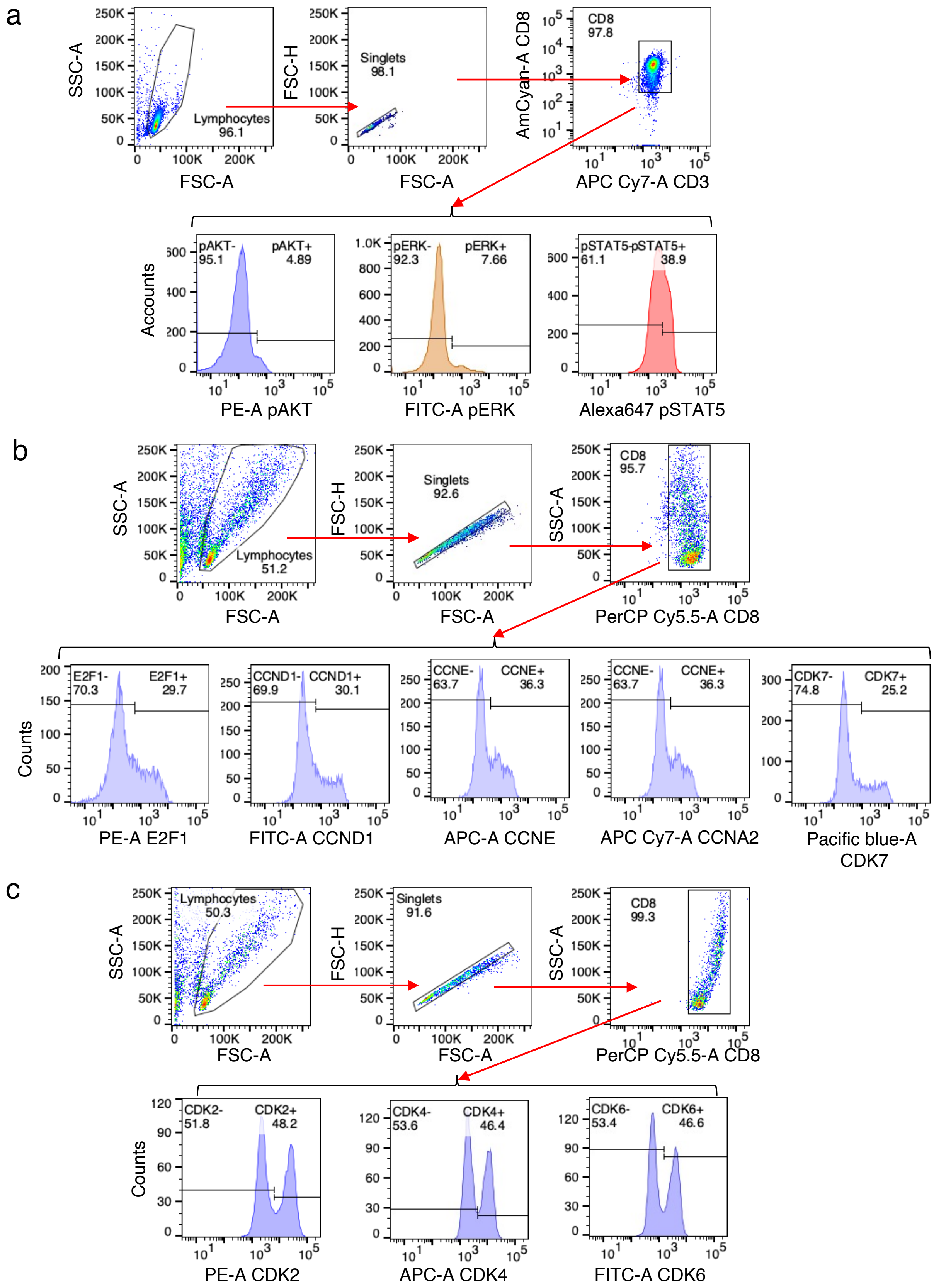

Supplementary Fig. 12. Flow cytometry gating strategies for IL-2-induced (**a**) pAKT, pERK, and pSTAT5 (for **Supplementary Fig. 4a, c, d, e**), (**b**) E2F1, CCND1, CCNE, CCNA2, and CDK7 (for **Supplementary Fig. 6a-e)**, and (**c**) CDK2, CDK4, and CDK6 in freshly isolated $CD8^+$ T cells (for **Supplementary Fig. 6f-h)**.

Supplementary Table 1. List of primers for the generation of mutant mice and genotyping. Four mutant residues in single-stranded oligonucleotides for generating *Stat5a* and *Stat5b* mutants are in red upper case. The residue for the Tyr to Phe change is also highlighted in bold. The 5’ PCR oligonucleotides were used for Sanger sequencing.

**Name**	**Sequence**
sgRNA for Stat5a	ccggcagcgaaagcagttga
sgRNA for Stat5b	ccggcagcgaaagcagctga
Single-stranded oligo for Stat5a	t gtg ctc att gcg ctt ccc ccc tac ccc cac ccc tgc ccg gca gcg aaG gcC gtC gac gga t**T**c gtg aag cca cag atc aag caa gtg gtc cct gag taa gtg tcc aga gcc agc tct ggg gct cc
Single-stranded oligo for Stat5b	ggg agg tgt ctc tga ggt ccc tgg ttt tca ctg ttc ttc tcc ccg gca gcg aaG gca gcA gaT gga t**T**c gtg aag cca cag atc aag cag gtg gtc ccc gag taa gtg tcc agg gcc cag ggc ggt agc tca cgg cca gca gt
5' PCR oligo for Stat5a	CGG AAG TCC TAG GGA AAT CG
3' PCR oligo for Stat5a	CAG TTG AAT CTT TCC TTC TCA AGG
5' PCR oligo for Stat5b	CAA GCC TCC CAG AAG ACA GA
3' PCR oligo for Stat5b	AGA GAC AGA CAC ACA GAG AGA GTC A

Supplementary Table 6. Antibodies for flow cytometry.

Antibody	Vender	Cat No	Clone	µl/10^6 cells
FITC CD122	BioLegend	123208	TM-β1	0.5 µl
FITC CD8a	BioLegend	100706	53-6.7	2 µl
FITC CD49b (DX5)	BioLegend	108906	DX5	2 µl
Alexa488 pERK (T202/Y204)	Cell Signaling	5315S	197G2	1:50
Alexa488 CD8a	BioLegend	100723	53-6.7	0.5 µl
PE CD3e	BioLegend	100308	145-2C11	1.25 µl
PE B220	BioLegend	103208	Ra3-6B2	1.25 µl
PE NK1.1	BioLegend	108707	PK1.36	1.25 µl
PE CD122	BioLegend	123210	TM-β1	1.25 µl
PE Foxp3	eBioSciences	12-5773-82	FJK-16s	1 µg/test
PE pRb (Ser807/811)	Cell Signaling	11917	D20B12	1:50
PE pAKT (pS473)	Cell Signaling	5315S	D9E	1:50
APC CD4	BioLegend	550351	RM4-5	2.5 µl
APC CD3e	BioLegend	100312	145-2C11	<5 µl
APC CD8a	BioLegend	100712	53-6.7	1.25 µl
APC CD122	BioLegend	123214	TM-β1	1.25 µl
APC CD335 (NKp46)	BioLegend	137608	29A1.4	<5 µl
APC CD49b (DX5)	BioLegend	108910	DX5	<1.25 µl
APC Streptavidin	BioLegend	405207	n/a	0.625 µl
Alexa647 pSTAT5 (Y694)	BD Biosciences	612599	47pSTAT5 (Y694)	20 µl
PE/Cy7 CD4	BioLegend	100422	GK1.5	1.25 µl
APC/Cy7 NK1.1	BioLegend	108724	PK1.36	≤ 5 µl
APC/Cy7 CD49d	BioLegend	103636	R1-2	≤ 5 µl
APC/Cy7 CD4	BioLegend	100414	GK1.5	5 µl
BV421 CD44	BioLegend	103039	IM7	≤ 1.25 µl
BV421 CD25	BioLegend	102034	PC61	0.5 µl
PerCP/Cy5.5 CD122	BioLegend	123212	TM-b1	≤ 2.5 µl
PerCP/Cy5.5CD25	BioLegend	101912	3C7	5 µl
Rb1	ThermoFisher	MA5-11387	1F8	5 µl
E2F1	ThermoFisher	MA5-32476	JJ092-02	0.5 µl
CDK7	ThermoFisher	MA5-32434	K6.83 (DCS-83)	0.5 µl
CCND1	ThermoFisher	MA5-14512	DCS-31	1.65 µl
CCNE	ThermoFisher	MA5-14336	JJ203-01	5 µl
CCNA2	ThermoFisher	MA5-32353	SP4	0.25 µl
CDK6	ThermoFisher	AHZ20232	HE12	0.25 µl
CDK2	ThermoFisher	MA5-17052	1A6	0.125 µl
CDK4	ThermoFisher	AHZ0202	SD2052	0.25 µl
Bioin TCRβ	BioLegend	109204	H57-597	0.5 µl

Bioin CD3	BioLegend	100304	145-2C11	0.5 µl
Bioin CD4	BioLegend	100404	GK1.5	0.5 µl
Bioin CD8a	BioLegend	100704	53-6.7	0.5 µl
Bioin CD19	BioLegend	152420	1D3	0.25 µl
Bioin IgM	BioLegend	406504	RMM-1	2 µl
Bioin Ter119	BioLegend	116204	Ter119	0.5 µl
Alexa647 Goat anti-Mouse IgG (H+L) Highly Cross-Adsorbed Secondary Antibody	ThermoFisher	A-21236	n/a	0.25 µl
Goat anti-Rabbit IgG (H+L), Superclonal™ Recombinant Secondary Antibody	ThermoFisher	A27040	n/a	0.5 µl
Alexa488 Goat anti-Mouse IgG (H+L), Superclonal™ Recombinant Secondary Antibody	ThermoFisher	A28175	n/a	0.5 µl
Goat anti-Rabbit IgG (H+L), Superclonal™ Recombinant Secondary Antibody	ThermoFisher	A27034	n/a	0.5 µl
PE F(ab')2-Goat anti-Mouse IgG (H+L) Secondary Antibody	eBioscience	12-4010-82	n/a	1 µl
F(ab')2-Goat anti-Rabbit IgG (H+L) Secondary Antibody	ThermoFisher	A10542	n/a	0.4 µl
Alexa405 Goat anti-Rabbit IgG (H+L) Cross-Adsorbed Secondary Antibody	ThermoFisher	A-31556	n/a	0.5 µl
Alexa750 Goat anti-Rabbit IgG (H+L) Cross-Adsorbed Secondary Antibody	ThermoFisher	A21039	n/a	0.5 µl

Supplementary Table 7. Antibodies for immunoprecipitation and western blotting.

Antibody	Vender	Cat No	Clone	Concentration/dilution
STAT5A	R&D	MAB2174	251619	2 μg/ml
STAT5B	R&D	MAB1584	389215	1 μg/ml
pSTAT5 (Y694)	Cell Signaling	9356	14H2	1:1000
IRDye 680RD donkey anti-mouse IgG	LI-COR	926-68072		1:25000
IRDye 800CW donkey anti-rabbit IgG	LI-COR	926-32213		1:25000
Myc	Cell Signaling	18583	E5Q6W	1:1000
β-Actin	Cell Signaling	3700	8H10D10	1:1000

Supplementary Table 8 Antibodies for ChIP-Seq.

Antibody	Vender	Cat No	Clone	Concentration/dilution
Rabbit IgG	Cell Signaling	3900S	DA1E	
STAT5A/STAT5B	Abcam	Ab194898	ERR16671-40	1:100
