# Supplementary material for: Tyrosine phosphorylation of both STAT5A and STAT5B is necessary for maximal IL-2 signaling and T cell proliferation
Source: Nat Commun. 2024 Aug 27;15:7372. doi: 10.1038/s41467-024-50925-6 (PMC11349758; doi:10.1038/s41467-024-50925-6)
Supplement: Supplementary file 3 — Description of Additional Supplementary Files [file 41467_2024_50925_MOESM3_ESM.pdf]

Description of Additional Supplementary Files

Supplementary Data Legends:

Supplementary Data 1. List of significantly regulated mRNAs identified by RNA-seq analysis in Stat5a and Sta5b WT CD8+ T cells in response to IL-2 stimulation.

Supplementary Data 2. List of mRNAs identified by RNA-seq analysis in IL-2-stimulated CD8+ T cells with altered expression in Stat5a KI vs WT and Stat5b KI vs WT.

Supplementary Data 3. List of genes whose expression and STAT5 binding were significantly diminished in Stat5a KI and Stat5b KI (CD8+ T cells in response to IL-2 stimulation.

Supplementary Data 4. List of proteins identified by mass spectrometric analysis in Stat5a WT and KI CD8+ T cells stimulated with IL-2.
