# Supplementary material for: Tyrosine phosphorylation of both STAT5A and STAT5B is necessary for maximal IL-2 signaling and T cell proliferation
Source: Nat Commun. 2024 Aug 27;15:7372. doi: 10.1038/s41467-024-50925-6 (PMC11349758; doi:10.1038/s41467-024-50925-6)
Supplement: Supplementary file 8 — Reporting Summary [file 41467_2024_50925_MOESM8_ESM.pdf]

nature portfolio

Corresponding author(s): Warren J. Leonard, Jian-Xin Lin

Last updated by author(s): May 13, 2024

Reporting Summary

Nature Portfolio wishes to improve the reproducibility of the work that we publish. This form provides structure for consistency and transparency in reporting. For further information on Nature Portfolio policies, see our Editorial Policies and the Editorial Policy Checklist.

Statistics

For all statistical analyses, confirm that the following items are present in the figure legend, table legend, main text, or Methods section.

n/a	Confirmed	
☐	☒	The exact sample size (n) for each experimental group/condition, given as a discrete number and unit of measurement
☐	☒	A statement on whether measurements were taken from distinct samples or whether the same sample was measured repeatedly
☐	☒	The statistical test(s) used AND whether they are one- or two-sided<br>*Only common tests should be described solely by name; describe more complex techniques in the Methods section.*
☒	☐	A description of all covariates tested
☒	☐	A description of any assumptions or corrections, such as tests of normality and adjustment for multiple comparisons
☐	☒	A full description of the statistical parameters including central tendency (e.g. means) or other basic estimates (e.g. regression coefficient) AND variation (e.g. standard deviation) or associated estimates of uncertainty (e.g. confidence intervals)
☐	☒	For null hypothesis testing, the test statistic (e.g. F, t, r) with confidence intervals, effect sizes, degrees of freedom and P value noted<br>*Give P values as exact values whenever suitable.*
☒	☐	For Bayesian analysis, information on the choice of priors and Markov chain Monte Carlo settings
☒	☐	For hierarchical and complex designs, identification of the appropriate level for tests and full reporting of outcomes
☒	☐	Estimates of effect sizes (e.g. Cohen's d, Pearson's r), indicating how they were calculated

Our web collection on statistics for biologists contains articles on many of the points above.

Software and code

Policy information about availability of computer code

Data collection	BD FACSDiva v8.01 (BD Bioscience) to acquire flow cytometry data on CANTO II and Odyssey CLx (LI-COR) to acquired western blot image.
Data analysis	Flowjo v10.10.0 (BD Biosciences) for FACS results.<br>GraphPad Prism v10 for statistics analysis.

For manuscripts utilizing custom algorithms or software that are central to the research but not yet described in published literature, software must be made available to editors and reviewers. We strongly encourage code deposition in a community repository (e.g. GitHub). See the Nature Portfolio guidelines for submitting code & software for further information.

Data

Policy information about availability of data

All manuscripts must include a data availability statement. This statement should provide the following information, where applicable:

- Accession codes, unique identifiers, or web links for publicly available datasets
- A description of any restrictions on data availability
- For clinical datasets or third party data, please ensure that the statement adheres to our policy

The authors declare that the data supporting the findings of this study are available within the manuscript and its Supplementary information. The RNA-seq and ChIP-seq data described in the manuscript have been deposited in the NCBI Gene Expression Omnibus (GEO) database and are accessible through the GEO.

SuperSeries access number GSE247343. The mass spectrometry data, the Raw files are uploaded with accession MSV000093320 and the FTP download link ftp://MSV000093320@massive.uusd.edu.

Research involving human participants, their data, or biological material

Policy information about studies with human participants or human data. See also policy information about sex, gender (identity/presentation), and sexual orientation and race, ethnicity and racism.

Reporting on sex and gender	N/A
Reporting on race, ethnicity, or other socially relevant groupings	N/A
Population characteristics	N/A
Recruitment	N/A
Ethics oversight	N/A

Note that full information on the approval of the study protocol must also be provided in the manuscript.

Field-specific reporting

Please select the one below that is the best fit for your research. If you are not sure, read the appropriate sections before making your selection.

☒ Life sciences ☐ Behavioural & social sciences ☐ Ecological, evolutionary & environmental sciences

For a reference copy of the document with all sections, see nature.com/documents/nr-reporting-summary-flat.pdf

Life sciences study design

All studies must disclose on these points even when the disclosure is negative.

Sample size	Sample sizes were selected based on those used in previous publications (Lin et al., Immunity 2012; Lin et al., Nature Communications 2017)
Data exclusions	No data were excluded.
Replication	All the experimental findings were reliably reproduced as validated by at least two biological replicates in at least two independent experiments unless otherwise indicated.
Randomization	Age- and sex-matched mice were used randomly to experimental and control groups.
Blinding	The investigators were not blinded to data collection and analysis. This approach is generally used for this type of studies because the genotype of the cells has to be predetermined before analysis.

Reporting for specific materials, systems and methods

We require information from authors about some types of materials, experimental systems and methods used in many studies. Here, indicate whether each material, system or method listed is relevant to your study. If you are not sure if a list item applies to your research, read the appropriate section before selecting a response.

Materials & experimental systems

n/a	Involved in the study	
☐	☒	Antibodies
☒	☐	Eukaryotic cell lines
☒	☐	Palaeontology and archaeology
☐	☒	Animals and other organisms
☒	☐	Clinical data
☒	☐	Dual use research of concern
☒	☐	Plants

Methods

n/a	Involved in the study	
☐	☒	ChIP-seq
☐	☒	Flow cytometry
☒	☐	MRI-based neuroimaging

Antibodies

Antibodies used

1. The following antibodies were used for cell culture: Anti-mouse CD3 (145-2C11, BioXCell), anti-mouse CD28 (1 g/ml, 37.51, BioXCell).

2. For flow cytometry: Biotin anti-mouse TCR beta (H57-597, 0.5 ul/10^6 cells), Biotin anti-mouse CD3e (145-2C11, 0.5 ul/10^6 cells), Biotin anti-mouse CD4 (GK1.5, 0.5 ul/10^6 cells), Biotin anti-mouse CD8a (53-6.7, 0.5 ul/10^6 cells), Biotin anti-mouse CD19 (1D3, 0.25 ul/10^6 cells), Biotin anti-mouse IgM (RMM-1, 2 ul/10^6 cells), Biotin anti-mouse Ter119 (Ter-119, 0.5 ul/10^6 cells), APC Streptavidin (0.625 ul/10^6 cells), B220 (RA3-6B2, 1.25 ul/10^6 cells), CD3e (145-2C11, ≤5 ul/10^6 cells), CD4 (GK1.5, 1.25 ul/10^6 cells), CD8a (53-6.7, 1.25 ul/10^6 cells), CD44 (IM7, 0.5 ul/10^6 cells), CD49d (R1-2, ≤2 ul/10^6 cells), CD25 (7D4, 5 ul/10^6 cells), CD25 (3C7, 5 ul/10^6 cells), CD25 (PC61, 1.25 ul/10^6 cell), CD122 (TM-b1, 0.5 ug/10^6 cells), CD49b (DX5, 0.5 ul/10^6 cells), CD49b (DX5, 1.25 ul/10^6 cells), NK1.1 (PK1.36, 5 ul/10^6 cells), CD335 (NKp46,29A1.4, 4 ul/10^6 cells), FOXP3 monoclonal antibody (FJK-16s, Invitrogen, 12-5773-82), FOXP3 (MF-14, 0.25 ul/10^6 cells), RB1 (1F8, 5 ul/10^6 cells), pRB (S807/811, D20B12, 1:400), E2F1 (JJ092-02, MA5-32476, 1:100), CCND1 (SP4, MA5-14512, 1:30), CCNA2 (SD2052, MA5-32353, 1:100), CCNE (HE12, MA5-14336, 1-2 ug/test), CDK2 (1A6, MA5-17052, 1:100), CDK4 (DCS-31, AHZ0202), CDK6 (K6.83 (DCS-83, AHZ0232), 1:100, CDK7 (JJ203-01, MA5-32434, 1:100), Alexa Fluor 405 Goat anti-Rabbit IgG (H+L, A31556, 1-10 ug/ml), Alexa Fluor 488 Goat anti-Rabbit IgG (Heavy chain, A27034, 1-5 ug/ml), Alexa Fluor 647 Goat anti-Rabbit IgG (Heavy chain, A27040, 1:50), Alexa Fluor 750 Goat anti-Rabbit IgG (H +L, A21039, 1-10 ug/ml), Alexa Fluor 488 Goat anti-Mouse IgG (H+L, A28175, 1-5 ug/ml), PE F(ab')2-Goat anti-Mouse IgG (H+L, 12-4010-82, 0.25 ug/test), Alexa Fluor 647 Goat anti-Mouse IgG (H+L, A21236, 1-10 ug/ml), pY694STAT5 (47/STAT5 pY694, BD, 612599, 20 ul/test), pERK (197G2, CST, 13214S, 1:50), and pAKT (D9E, CST, 5315S, 1:50).

3. For immunoprecipitation and western blotting: rabbit anti-STAT5A (1216), rabbit anti-STAT5B (1219), mouse monoclonal anti-STAT5A (251619, R&D, MAB2174, 2 ug/ml), anti-STAT5B (389215, R&D, MAB1584, 1 ug/ml), and anti-pYSTAT5 antibodies (14H2, CST, 9356, 1:1000), IRDye 680RD donkey anti-mouse IgG secondary antibody (LI-COR Biosciences, 926-68072, 1:20000-25000), IRDye 800CW donkey anti-rabbit IgG secondary antibody (LI-COR Biosciences, 926-32213, 1:20000-25000), rabbit monoclonal anti-Myc (E5Q6W, CST, 18583, 1:1000) and mouse monoclonal anti-beta-actin (8H10D10, CST, 3700, 1:1000) antibodies.

4. For ChIP-seq: rabbit IgG (DA1E, Cell Signaling Technology, 3900S) and anti-STAT5A/B antibody (Ab194898, Abcam, 1:100),

Validation

1. The following antibodies for cell culture have been validated for the specificity and application by the manufacturer (see detailed reference on the corresponding websites).
anti-mouse CD3e: https://bioxcell.com/invivomab-anti-mouse-cd3e-be0001-1
anti-mouse CD28: https://bioxcell.com/invivomab-anti-mouse-cd28-be0015-1

2. The following antibodies for flow cytometry were validated for the specificity and application by the manufacturers (see detailed references on their websites).

Live/DEAD Fixable Aqua Dead Cell Stain Kit, https://www.thermofisher.com/order/catalog/product/L34957
B220 (RA3-6B2), https://www.biolegend.com/en-us/products/pe-anti-mouse-human-cd45r-b220-antibody-4477
CD3e (145-2C11), https://www.biolegend.com/en-us/products/apc-anti-mouse-cd3epsilon-antibody-21
CD4 (GK1.5), https://www.biolegend.com/en-us/products/pe-anti-mouse-cd4-antibody-250
CD8a (53-6.7), https://www.biolegend.com/en-us/products/apc-anti-mouse-cd8a-antibody-150
CD44 (IM7), https://www.biolegend.com/en-us/products/pacific-blue-anti-mouse-human-cd44-antibody-3099
CD49d (R1-2), https://www.biolegend.com/en-us/products/fitc-anti-mouse-cd49d-antibody-438
CD25 (3C7), https://www.biolegend.com/en-us/products/percp-cyanine5-5-anti-mouse-cd25-antibody-6667
CD25 (PC61), https://www.biolegend.com/en-us/products/apc-anti-mouse-cd25-antibody-420
CD122 (TM-b1), https://www.biolegend.com/en-us/products/percp-cyanine5-5-anti-mouse-cd122-il-2rbeta-antibody-8873
NK1.1 (PK1.36), https://www.biolegend.com/en-us/products/apc-cyanine7-anti-mouse-nk-1-1-antibody-4002
CD49b (DX5), https://www.biolegend.com/en-us/products/fitc-anti-mouse-cd49b-pan-nk-cells-antibody-233
CD335 (NKp46), https://www.biolegend.com/en-us/products/apc-anti-mouse-cd335-nkp46-antibody-6676
FOXP3 monoclonal antibody (FJK-16s, 12-5773-82), https://www.thermofisher.com/antibody/product/FOXP3-Antibody-clone-FJK-16s-Monoclonal/12-5773-82
FOXP3 (MF-14), https://www.biolegend.com/en-us/products/alexa-fluor-647-anti-mouse-foxp3-antibody-4662
TCR beta (H57-597), https://www.biolegend.com/en-us/products/biotin-anti-mouse-tcr-beta-chain-antibody-269
CD3 (145-2C11), https://www.biolegend.com/en-us/products/biotin-anti-mouse-cd3epsilon-antibody-22
CD4 (GK1.5), https://www.biolegend.com/en-us/products/biotin-anti-mouse-cd4-antibody-247
CD8a (53-6.7), https://www.biolegend.com/en-us/products/biotin-anti-mouse-cd8a-antibody-152
CD19 (1D3), https://www.biolegend.com/en-us/products/biotin-anti-mouse-cd19-antibody-22866
IgM (RMM-1), https://www.biolegend.com/en-us/products/biotin-anti-mouse-igm-2333
Ter119 (Ter-119), https://www.biolegend.com/en-us/products/biotin-anti-mouse-ter-119-erythroid-cells-antibody-1864
Streptavidin, https://www.biolegend.com/en-us/products/apc-streptavidin-1470
RB1 (1F8), https://www.thermofisher.com/antibody/product/Rb1-Tumor-Suppressor-Protein-Antibody-clone-1F8-Monoclonal/5925-MSM1-P0
pRB (S807/811, D20B12), https://www.cellsignal.com/products/primary-antibodies/phospho-rb-ser807-811-d20b12-xp-rabbit-mab/8516
E2F1 (JJ092-02, MA5-32476), https://www.thermofisher.com/antibody/product/E2F1-Antibody-clone-JJ092-02-Recombinant-Monoclonal/MA5-32476
CCND1 (SP4, MA5-14512), https://www.thermofisher.com/antibody/product/Cyclin-D1-Antibody-clone-SP4-Recombinant-Monoclonal/MA5-14512
CCNA2 (SD2052, MA5-32353), https://www.thermofisher.com/antibody/product/Cyclin-A2-Antibody-clone-SD2052-Recombinant-Monoclonal/MA5-32353
CCNE (HE12, MA5-14336), https://www.thermofisher.com/antibody/product/Cyclin-E-Antibody-clone-HE12-Monoclonal/MA5-14336
CDK2 (1A6, MA5-17052), https://www.thermofisher.com/antibody/product/CDK2-Antibody-clone-1A6-Monoclonal/MA5-17052
CDK4 (DCS-31, AHZ0202), https://www.thermofisher.com/antibody/product/CDK4-Antibody-clone-DCS-31-Monoclonal/AHZ0202

CDK6 (K6.83 (DCS-83), AHZ0232), https://www.thermofisher.com/antibody/product/CDK6-Antibody-clone-K6-83-DCS-83-Monoclonal/AHZ0232
CDK7 (JJ203-01, MA5-32434), https://www.thermofisher.com/antibody/product/CDK7-Antibody-clone-JJ203-01-Recombinant-Monoclonal/MA5-32434
Alexa Fluor 405 Goat anti-Rabbit IgG (H+L, A31556), https://www.thermofisher.com/antibody/product/Goat-anti-Rabbit-IgG-H-L-Cross-Adsorbed-Secondary-Antibody-Polyclonal/A-31556
Alexa Fluor 488 Goat anti-Rabbit IgG (Heavy chain, A27034), https://www.thermofisher.com/antibody/product/Goat-anti-Rabbit-IgG-Heavy-chain-Secondary-Antibody-Recombinant-Polyclonal/A27034
Alexa Fluor 647 Goat anti-Rabbit IgG (Heavy chain, A27040), https://www.thermofisher.com/antibody/product/Goat-anti-Rabbit-IgG-Heavy-chain-Secondary-Antibody-Recombinant-Polyclonal/A27040
Alexa Fluor 750 Goat anti-Rabbit IgG (H+L, A21039), https://www.thermofisher.com/antibody/product/Goat-anti-Rabbit-IgG-H-L-Cross-Adsorbed-Secondary-Antibody-Polyclonal/A-21039
Alexa Fluor 488 Goat anti-Mouse IgG (H+L, A28175), https://www.thermofisher.com/antibody/product/Goat-anti-Mouse-IgG-H-L-Secondary-Antibody-Recombinant-Polyclonal/A28175
PE F(ab')2-Goat anti-Mouse IgG (H+L, 12-4010-82), https://www.thermofisher.com/antibody/product/Goat-anti-Mouse-IgG-H-L-Secondary-Antibody-Polyclonal/12-4010-82#:~:text=Description%3A%20The%20goat%20anti%2Dmouse,hamster%2C%20and%20rabbit%20serum%20proteins.
Alexa Fluor 647 Goat anti-Mouse IgG (H+L, A21236), https://www.thermofisher.com/antibody/product/Goat-anti-Mouse-IgG-H-L-Highly-Cross-Adsorbed-Secondary-Antibody-Polyclonal/A-21236
pY694STAT5 (47/STAT5 pY694, BD, 612599), https://www.bdbiosciences.com/en-us/products/reagents/flow-cytometry-reagents/research-reagents/single-color-antibodies-ruo/alexa-fluor-647-mouse-anti-stat5-py694.612599
pERK (197G2, CST, 13214S), https://www.cellsignal.com/products/antibody-conjugates/phospho-p44-42-mapk-erk1-2-thr202-tyr204-197g2-rabbit-mab-alexa-fluor-488-conjugate/13214
pAKT (D9E, CST, 5315S), https://www.cellsignal.com/products/antibody-conjugates/phospho-akt-ser473-d9e-xp-rabbit-mab-pe-conjugate/5315

3. The following antibodies for immunoprecipitation and western blotting were validated for the specificity and application by the manufacturers (see detailed references on their websites).

rabbit anti-STAT5A (1216), DOI:https://doi.org/10.1074/jbc.271.18.10738
rabbit anti-STAT5B (1219), DOI:https://doi.org/10.1074/jbc.271.18.10738
mouse monoclonal anti-STAT5A (251619, R&D, MAB2174), https://www.rndsystems.com/products/human-mouse-stat5a-antibody-251619_mab2174
anti-STAT5B (389215, R&D, MAB1584), https://www.rndsystems.com/products/human-stat5b-antibody-389215_mab1584
anti-pYSTAT5 antibodies (14H2, CST, 9356), https://www.cellsignal.com/products/primary-antibodies/phospho-stat5-tyr694-14h2-mouse-mab/9356
IRDye 680RD donkey anti-mouse IgG secondary antibody (LI-COR Biosciences, 926-68072), https://www.licor.com/bio/reagents/irdye-680rd-donkey-anti-mouse-igg-secondary-antibody
IRDye 800CW donkey anti-rabbit IgG secondary antibody (LI-COR Biosciences, 926-32213), https://www.licor.com/bio/reagents/irdye-800cw-donkey-anti-rabbit-igg-secondary-antibody
rabbit monoclonal anti-Myc (E5Q6W, CST, 18583), https://www.cellsignal.com/products/primary-antibodies/c-myc-e5q6w-rabbit-mab/18583
mouse monoclonal anti-beta-actin (CST, 3700) antibody, https://www.cellsignal.com/products/primary-antibodies/b-actin-8h10d10-mouse-mab/3700

4. The following antibody for ChIP-seq was validated for specificity by the manufacturer and for ChIP-seq application by this study.

anti-STAT5A/B antibody (Ab194898, Abcam), https://www.abcam.com/products/primary-antibodies/stat5a--stat5b-antibody-epr16671-40-ab194898.html
rabbit IgG (3900S, Cell Signaling Technology), https://www.cellsignal.com/products/primary-antibodies/rabbit-da1e-mab-igg-xp-174-isotype-control/3900

Animals and other research organisms

Policy information about studies involving animals; ARRIVE guidelines recommended for reporting animal research, and Sex and Gender in Research

Laboratory animals	Tyrosine mutant knock-in mouse lines for Stat5a, Stat5b, and Stat5a/b were generated using CRISPR-Cas9 method by the NHLBI Transgenic Core, the founder mice were identified by PCR and Sanger sequencing. The heterozygous founders were then back-crossed with C57/BL6J for six generations before being used for experiments. The mice were housed in specific pathogen-free mouse facilities at the National Institutes of Health. Mice were housed at 72oF +/- 3oF with humidity at an average of 50% and a dark/light cycle of 12hr/12hr.
Wild animals	No wild mice were used in this study.
Reporting on sex	Both male and female mice were included in all experiments reported in this manuscript and no statistical differences between genders were observed in immune phenotypes reported in this study.
Field-collected samples	No samples were collected from the field.
Ethics oversight	All mouse protocols were approved by the National Heart, Lung and Blood Institute Animal Care Use Committee, and experiments followed NIH guidelines for using animals in intramural research.

Note that full information on the approval of the study protocol must also be provided in the manuscript.

Plants

Seed stocks	N/A
Novel plant genotypes	N/A
Authentication	N/A

ChIP-seq

Data deposition

☒ Confirm that both raw and final processed data have been deposited in a public database such as GEO.

☒ Confirm that you have deposited or provided access to graph files (e.g. BED files) for the called peaks.

Data access links
May remain private before publication.

https://www.ncbi.nlm.nih.gov/geo/query/acc.cgi?acc=GSE247343
Reviewer token: ohmdogwcbfktdmz

Files in database submission

GSM7887458 RNASeq STAT5A.0h.WT rep1
GSM7887459 RNASeq STAT5A.0h.WT rep2
GSM7887460 RNASeq STAT5A.0h.WT rep3
GSM7887461 RNASeq STAT5A.0h.KI rep1
GSM7887462 RNASeq STAT5A.0h.KI rep2
GSM7887463 RNASeq STAT5A.0h.KI rep3
GSM7887464 RNASeq STAT5A.4h.WT rep1
GSM7887465 RNASeq STAT5A.4h.WT rep2
GSM7887466 RNASeq STAT5A.4h.WT rep3
GSM7887467 RNASeq STAT5A.4h.KI rep1
GSM7887468 RNASeq STAT5A.4h.KI rep2
GSM7887469 RNASeq STAT5A.4h.KI rep3
GSM7887470 RNASeq STAT5A.24h.WT rep1
GSM7887471 RNASeq STAT5A.24h.WT rep2
GSM7887472 RNASeq STAT5A.24h.WT rep3
GSM7887473 RNASeq STAT5A.24h.KI rep1
GSM7887474 RNASeq STAT5A.24h.KI rep2
GSM7887475 RNASeq STAT5A.24h.KI rep3
GSM7887476 RNASeq STAT5A.48h.WT rep1
GSM7887477 RNASeq STAT5A.48h.WT rep2
GSM7887478 RNASeq STAT5A.48h.WT rep3
GSM7887479 RNASeq STAT5A.48h.KI rep1
GSM7887480 RNASeq STAT5A.48h.KI rep2
GSM7887481 RNASeq STAT5A.48h.KI rep3
GSM7887482 RNASeq STAT5B.0h.WT rep1
GSM7887483 RNASeq STAT5B.0h.WT rep2
GSM7887484 RNASeq STAT5B.0h.WT rep3
GSM7887485 RNASeq STAT5B.0h.KI rep1
GSM7887486 RNASeq STAT5B.0h.KI rep2
GSM7887487 RNASeq STAT5B.0h.KI rep3
GSM7887488 RNASeq STAT5B.4h.WT rep1
GSM7887489 RNASeq STAT5B.4h.WT rep2
GSM7887490 RNASeq STAT5B.4h.WT rep3
GSM7887491 RNASeq STAT5B.4h.KI rep1
GSM7887492 RNASeq STAT5B.4h.KI rep2
GSM7887493 RNASeq STAT5B.4h.KI rep3
GSM7887494 RNASeq STAT5B.24h.WT rep1
GSM7887495 RNASeq STAT5B.24h.WT rep2
GSM7887496 RNASeq STAT5B.24h.WT rep3
GSM7887497 RNASeq STAT5B.24h.KI rep1
GSM7887498 RNASeq STAT5B.24h.KI rep2
GSM7887499 RNASeq STAT5B.24h.KI rep3
GSM7887500 RNASeq STAT5B.48h.WT rep1
GSM7887501 RNASeq STAT5B.48h.WT rep2
GSM7887502 RNASeq STAT5B.48h.WT rep3
GSM7887503 RNASeq STAT5B.48h.KI rep1
GSM7887504 RNASeq STAT5B.48h.KI rep2

GSM7887505 RNASeq STAT5B.48h.KI rep3
GSM7887506 ChIPSeq 5aWT IL2 IgG control
GSM7887507 ChIPSeq 5aWT Cont STAT5
GSM7887508 ChIPSeq 5aWT IL2 STAT5
GSM7887509 ChIPSeq 5aKI Cont STAT5
GSM7887510 ChIPSeq 5aKI IL2 STAT5
GSM7887511 ChIPSeq 5bWT Cont STAT5
GSM7887512 ChIPSeq 5bWT IL2 STAT5
GSM7887513 ChIPSeq 5bKI Cont STAT5
GSM7887514 ChIPSeq 5bKI IL2 STAT5

Genome browser session
(e.g. UCSC)

https://www.igv.org/
with local .tdf files

Methodology

Replicates

3 replicates for RNA-Seq and one replicate for ChIPSeq

Sequencing depth

Libraries Descriptions Raw Mapped read length single or paired-end
GSM7887458 RNASeq STAT5A.0h.WT rep1 61144902 58351872 50 single
GSM7887459 RNASeq STAT5A.0h.WT rep2 62499744 59511632 50 single
GSM7887460 RNASeq STAT5A.0h.WT rep3 87818916 83708853 50 single
GSM7887461 RNASeq STAT5A.0h.KI rep1 42995441 40828629 50 single
GSM7887462 RNASeq STAT5A.0h.KI rep2 63651549 674328 50 single
GSM7887463 RNASeq STAT5A.0h.KI rep3 51509783 49224219 50 single
GSM7887464 RNASeq STAT5A.4h.WT rep1 62468604 59045306 50 single
GSM7887465 RNASeq STAT5A.4h.WT rep2 43902221 38715604 50 single
GSM7887466 RNASeq STAT5A.4h.WT rep3 58755303 55640771 50 single
GSM7887467 RNASeq STAT5A.4h.KI rep1 57300454 53990310 50 single
GSM7887468 RNASeq STAT5A.4h.KI rep2 71379359 67257077 50 single
GSM7887469 RNASeq STAT5A.4h.KI rep3 21312823 18122792 50 single
GSM7887470 RNASeq STAT5A.24h.WT rep1 51325962 49368282 50 single
GSM7887471 RNASeq STAT5A.24h.WT rep2 54953146 52698066 50 single
GSM7887472 RNASeq STAT5A.24h.WT rep3 62159507 59745235 50 single
GSM7887473 RNASeq STAT5A.24h.KI rep1 55432984 53021338 50 single
GSM7887474 RNASeq STAT5A.24h.KI rep2 56836996 54391936 50 single
GSM7887475 RNASeq STAT5A.24h.KI rep3 52579410 50359009 50 single
GSM7887476 RNASeq STAT5A.48h.WT rep1 65384213 63085529 50 single
GSM7887477 RNASeq STAT5A.48h.WT rep2 59802354 57890477 50 single
GSM7887478 RNASeq STAT5A.48h.WT rep3 71072395 68550921 50 single
GSM7887479 RNASeq STAT5A.48h.KI rep1 62211661 59880800 50 single
GSM7887480 RNASeq STAT5A.48h.KI rep2 65320454 62679536 50 single
GSM7887481 RNASeq STAT5A.48h.KI rep3 62550644 60156065 50 single
GSM7887482 RNASeq STAT5B.0h.WT rep1 57847169 55348424 50 single
GSM7887483 RNASeq STAT5B.0h.WT rep2 57744696 55363588 50 single
GSM7887484 RNASeq STAT5B.0h.WT rep3 56773042 54296243 50 single
GSM7887485 RNASeq STAT5B.0h.KI rep1 57648068 54844622 50 single
GSM7887486 RNASeq STAT5B.0h.KI rep2 55023079 52576293 50 single
GSM7887487 RNASeq STAT5B.0h.KI rep3 53550459 50805154 50 single
GSM7887488 RNASeq STAT5B.4h.WT rep1 64557463 61387605 50 single
GSM7887489 RNASeq STAT5B.4h.WT rep2 59050789 56138804 50 single
GSM7887490 RNASeq STAT5B.4h.WT rep3 51357351 48976435 50 single
GSM7887491 RNASeq STAT5B.4h.KI rep1 58786148 55717815 50 single
GSM7887492 RNASeq STAT5B.4h.KI rep2 57805106 54773288 50 single
GSM7887493 RNASeq STAT5B.4h.KI rep3 58256890 54945383 50 single
GSM7887494 RNASeq STAT5B.24h.WT rep1 65326801 62842062 50 single
GSM7887495 RNASeq STAT5B.24h.WT rep2 60943329 58602379 50 single
GSM7887496 RNASeq STAT5B.24h.WT rep3 60380570 58093093 50 single
GSM7887497 RNASeq STAT5B.24h.KI rep1 59095455 56146942 50 single
GSM7887498 RNASeq STAT5B.24h.KI rep2 52301359 49736662 50 single
GSM7887499 RNASeq STAT5B.24h.KI rep3 57298880 54628986 50 single
GSM7887500 RNASeq STAT5B.48h.WT rep1 64706713 62657215 50 single
GSM7887501 RNASeq STAT5B.48h.WT rep2 60247064 58375446 50 single
GSM7887502 RNASeq STAT5B.48h.WT rep3 55648516 53878046 50 single
GSM7887503 RNASeq STAT5B.48h.KI rep1 53042738 50786398 50 single
GSM7887504 RNASeq STAT5B.48h.KI rep2 57625701 55111346 50 single
GSM7887505 RNASeq STAT5B.48h.KI rep3 56568713 53907884 50 single
GSM7887506 ChIPSeq 5aWT IL2 IgG control 55400680 44170033 50 single
GSM7887507 ChIPSeq 5aWT Cont STAT5 51515747 46309324 50 single
GSM7887508 ChIPSeq 5aWT IL2 STAT5 51631296 49850486 50 single
GSM7887509 ChIPSeq 5aKI Cont STAT5 40930191 36068384 50 single
GSM7887510 ChIPSeq 5aKI IL2 STAT5 46406005 43689225 50 single
GSM7887511 ChIPSeq 5bWT Cont STAT5 48811154 41643742 50 single
GSM7887512 ChIPSeq 5bWT IL2 STAT5 52718252 47035543 50 single

	GSM7887513 ChIPSeq 5bKI Cont STAT5 44349122 38463668 50 single<br>GSM7887514 ChIPSeq 5bKI IL2 STAT5 51808905 45254145 50 single
Antibodies	anti-STAT5A/B antibody (Ab194898, Abcam), https://www.abcam.com/products/primary-antibodies/stat5a--stat5b-antibody-epr16671-40-ab194898.html<br>rabbit IgG (3900S, Cell Signaling Technology), https://www.cellsignal.com/products/primary-antibodies/rabbit-da1e-mab-igg-xp-174-isotype-control/3900
Peak calling parameters	-g 'mm' -p 1e-5 -m 10,30 -shiftsize=100 --keep-dup=1 --verbose=0 --diag
Data quality	Raw FASTQ files were assessed with FastQC v0.11.9; Sequenced reads were aligned to the mm10 genome assembly using Bowtie 2.2.6 and Tophat 2.0.11; Aligned reads are converted to BAM files using samtools v0.1.8. BAM files are subsequently converted to BED files using bedtools v2.25.0. MACS is used to call peaks using the BED files. R package edgeR was used to identify differentially expressed genes.
Software	FastQC v0.11.9;bowtie v2.2.6; Tophat v2.0.11; samtools v0.1.8; bedtools v2.25.0; igvtools v2.3.82; macs v1.4.2; meme v5.4.1; R v4.3.0; edgeR v3.42.2

Flow Cytometry

Plots

Confirm that:

☒ The axis labels state the marker and fluorochrome used (e.g. CD4-FITC).

☒ The axis scales are clearly visible. Include numbers along axes only for bottom left plot of group (a 'group' is an analysis of identical markers).

☒ All plots are contour plots with outliers or pseudocolor plots.

☒ A numerical value for number of cells or percentage (with statistics) is provided.

Methodology

Sample preparation	For flow cytometry analysis of cellularity, spleens, thymi, and bone marrow were gently ground under 40 um nylon mesh using the flat end of a 3-ml syringes. Red blood cells were removed using ACK lysis buffer, followed by washing the cells with RPMI1640 medium. To purify splenic total T cells or CD8+ T cells, red blood cells were not lyzed by ACK and purified using EasySep Mouse T cell Isolation Kit (STEMCell Technologies, 19851) and the EasySep Mouse CD8+ T cell Isolation Kit (STEMCell Technologies, 19853), respectively.
Instrument	FACSCANTO II (BD Biosciences)
Software	BD FACSDiva software was used to acquire flow cytometry data on FACSCANTO II cytometer (BD Biosciences) and Flowjo v10.10.0 was used to analyze FACS results.
Cell population abundance	The purity of mouse spleen T cells or CD8+ T cells was about 95%.
Gating strategy	For all experiments, FSC-A vs. SSC-A gates was used to identify lymphocytes, singlets were gated by FSC-A vs. FSC-H, and live cells were those negatively stained using LIVE/DEAD FIXABLE Aqua Dead Cell Stain kit. T cells were gated by anti-mouse CD3 antibody, CD8 T cells were gated by anti-mouse CD3 and anti-mouse CD8 antibodies, CD8 T cells were further gated by anti-mouse CD44, anti-mouse CD122, and anti-mouse CD49d antibodies, CD4 T cells were gated by anti-mouse CD3 and anti-mouse CD4 antibodies, CD4 T cells were further gated by anti-mouse CD25 and anti-mouse FOXP3 antibodies, bone marrow NK cells were gated by lineage markers Bitoin anti-mouse TCRb, Bitoin anti-mouse CD3, Bitoin anti-mouse CD4, Bitoin anti-mouse CD8a, Bitoin anti-mouse CD19, Bitoin anti-mouse IgM, Bitoin anti-mouse Ter119 antibodies and Streptavidin-APC, then by anti-mouse CD122, anti-mouse NK1.1 and anti-mouse CD49b (DX5) antibodies, spleen NK cells were gated by anti-mouse CD3, anti-mouse NK1.1, anti-mouse CD122, or anti-mouse CD49b (DX5), or anti-mouse CD335 (NKp46) antibodies.

☐ Tick this box to confirm that a figure exemplifying the gating strategy is provided in the Supplementary Information.
